# A *Trans*-Acting Protein Effect Causes Severe Eye Malformation in the *Mp* Mouse

**DOI:** 10.1371/journal.pgen.1003998

**Published:** 2013-12-12

**Authors:** Joe Rainger, Margaret Keighren, Douglas R. Keene, Noe L. Charbonneau, Jacqueline K. Rainger, Malcolm Fisher, Sebastien Mella, Jeffrey T-J. Huang, Lorraine Rose, Rob van't Hof, Lynne Y. Sakai, Ian J. Jackson, David R. FitzPatrick

**Affiliations:** 1 The MRC Human Genetics Unit, MRC Institute of Genetic and Molecular Medicine, University of Edinburgh, Western General Hospital, Edinburgh, United Kingdom; 2 Shriners Hospital for Children, Portland, Oregon, United States of America; 3 Biomarker and Drug Analysis Core Facility, Medical Research Institute, School of Medicine, University of Dundee, Dundee, United Kingdom; 4 Molecular Medicine Centre, MRC Institute of Genetic and Molecular Medicine, University of Edinburgh, Western General Hospital, Edinburgh, United Kingdom; The University of Hong Kong, Hong Kong

## Abstract

*Mp* is an irradiation-induced mouse mutation associated with microphthalmia, micropinna and hind limb syndactyly. We show that *Mp* is caused by a 660 kb balanced inversion on chromosome 18 producing reciprocal 3-prime gene fusion events involving *Fbn2* and *Isoc1*. The *Isoc1-Fbn2* fusion gene (*Isoc1^Mp^*) mRNA has a frameshift and early stop codon resulting in nonsense mediated decay. Homozygous deletions of *Isoc1* do not support a significant developmental role for this gene. The *Fbn2-Isoc1* fusion gene (*Fbn2* ^Mp^) predicted protein consists of the N-terminal Fibrillin-2 (amino acids 1–2646, exons 1–62) lacking the C-terminal furin-cleavage site with a short out-of-frame extension encoded by the final exon of *Isoc1*. The *Mp* limb phenotype is consistent with that reported in *Fbn2* null embryos. However, severe eye malformations, a defining feature of *Mp*, are not seen in *Fbn2* null animals. Fibrillin-2^Mp^ forms large fibrillar structures within the rough endoplasmic reticulum (rER) associated with an unfolded protein response and quantitative mass spectrometry shows a generalised defect in protein secretion in conditioned media from mutant cells. In the embryonic eye *Fbn2* is expressed within the peripheral ciliary margin (CM). *Mp* embryos show reduced canonical Wnt-signalling in the CM – known to be essential for ciliary body development - and show subsequent aplasia of CM-derived structures. We propose that the *Mp* “worse-than-null” eye phenotype plausibly results from a failure in normal trafficking of proteins that are co-expressed with *Fbn2* within the CM. The prediction of similar trans-acting protein effects will be an important challenge in the medical interpretation of human mutations from whole exome sequencing.

## Author Summary

With the current increase in large-scale sequencing efforts, correct interpretation of mutation consequences has never been more important. Here, we present evidence for a *trans*-acting protein effect in a novel mutation of *Fbn2*, associated with severe developmental eye defects not found in loss of function Fibrillin-2 alleles. The mutant protein is expressed in the developing eye but is unable to exit the cells, instead forming large protein aggregates within the endoplasmic reticulum. We observed ER-stress in mutant eyes, and detected a general reduction to secretion of co-expressed proteins in cell cultures. We propose that similar effects could be caused by mutations to other proteins that are trafficked through the ER, highlighting a disease mechanism that results in different clinical outcomes than observed, or predicted, from loss-off-function alleles.

## Introduction

The accurate prediction of the phenotypic consequences of individual mutations is of increasing medical importance with the rapid development of whole genome sequencing technologies. Different mutations can have distinct effects on expression of the gene, its function, or localisation of its product. It is therefore not surprising that allelic heterogeneity can result in multiple distinct disorders from mutations in a single gene (e.g. *LMNA* [1], *FLNA* [2], *FGFR1* [3]). Such heterogeneity, in combination with stochastic effects and genetic and/or environmental modifiers, also accounts for phenotypic variability within an individual disorder.

Particular mutations cause disease due to a failure of normal post-translational processing and protein folding. For example, the mutation-specific induction of endoplasmic reticulum (ER) stress responses seen in disorders including late onset neurodegenerative diseases [4], congenital hearing loss [5] and skeletal dysplasias [6]. The mechanisms proposed for pathogeneisis associated with ER stress include: loss of function of the mutant protein [7], alteration of the cell specific differentiation state [6] or uncharacterised generalised cytopathy [4].

Anophthalmia (absence of the eye) and microphthalmia (small eye) are important causes of congenital visual impairments in developed countries with a live birth prevalence rate of 0.2 and 1.7 per 10,000 live births respectively [8], [9]. In most cases the cause of anophthalmia/microphthalmia remains unknown [10] although several single gene causes have been discovered including *SOX2* [11], [12], *OTX2* [13], *STRA6* [14], *FOXE3* [15], *SMOC1* [16], [17], [18] and *PAX6* [19]. Of these, heterozygous loss-of-function mutations in *SOX2* are the most common, accounting for 20–40% of bilaterally affected cases [20]. SOX2 functions at multiple stages during eye development, including during lens induction [21], [22] and formation and maintenance of the ciliary margin (CM) at the distal peripheral rim of the optic cup [23]. The CM forms the ciliary epithelium and the inner layer of the iris and controls ciliary muscle and stroma development [24]. At the boundary of the CM and the neural retina (NR), multipotent cells make a binary decision to commit to either CM or NR fate [23]. *SOX2* expression, which marks the boundary between CM and NR, is dependent on canonical Wnt-signaling [25], [26]. Loss of either Wnt-signalling or *SOX2* expression in the CM leads to failure of the development of the ciliary body structures and thinning and rosetting of the neural retina [23], [27], [28].

The *Mp* mouse was generated through the irradiation mutagenesis programme at the Oak Ridge National Laboratory in the 1960's. *Mp* homozygotes appeared to be anophthalmic and to have syndactylyyly of the hindlimbs [29]. Here, we identify the cause of *Mp* as a balanced 660 kb inversion on chromosome 18. The consequent gene fusion events result in the production of a C-terminally truncated fibrillin-2 protein that is retained in the rough-ER(rER) of expressing cells. Null mutations of *Fbn2* accurately phenocopy the *Mp* limb anomalies but are not associated with any ocular malformation [30], [31], [32]. Fbn2^Mp^ inclusions trigger the unfolded protein response (UPR) in a subset of cells within the CM resulting in aplasia of the ciliary apparatus with thinning and rosetting of the neural retina. The UPR seen in *Mp* is associated with signalling and patterning anomalies and a reduction in general protein secretion in Fbn2^Mp^ expressing cells. This mechanism has broader significance in the interpretation of human disease-causing mutations. It suggests that the phenotypic effect of specific UPR-inducing mutations may not result from loss of that gene product but rather from loss of co-expressed gene products that are also processed through the ER.

## Results

### Main adult phenotypes of *Mp*

**Figure 1:**
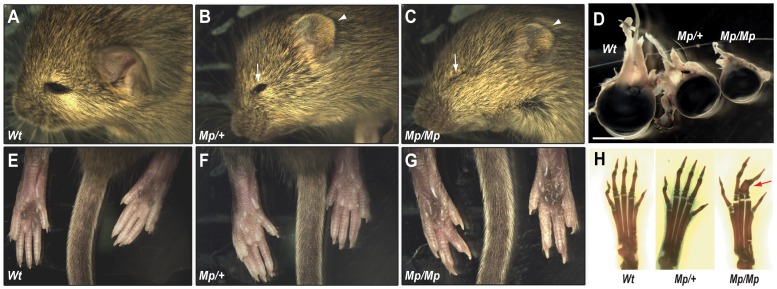
The *Mp* phenotype was characterised by severe ocular and limb abnormalities. (A–C) Heterozygous (*Mp/+*) animals had apparent microphthalmia (arrow) and reduced ear size (arrowhead) compared to Wt. Homozygotes (*Mp/Mp*) were more severely affected with closed eyes, indicating anophthalmia (arrow), and further reduced ear size (arrowhead). (D) Dissection of adult eyes revealed microphthalmia in both *Mp* genotypes and revealed a graded reduction in size compared to Wt (scale bar = 3.0 mm). (E–G) Hindlimb syndactylyyly was identified in *Mp/Mp* animals with the presence of only 4 digits on each paw compared to Wt and *Mp/+*. (H) Skeletal analysis revealed the presence of all 5 complete digital rays in Wt and *Mp/+* hindlimbs but revealed osseous fusions of phalanges (arrow) within *Mp/Mp* mid-axial digits (digits 2–4).

We bred *Mp* onto both the C57BL/6J inbred, and the CD1 outbred background strains. The phenotype in these mice was consistent with that documented in the previous report ([29]; Figure 1A–H), with small eyes and ears in adult heterozygotes (Figure 1B; *Mp/+*) and small ears, apparent anophthalmia and hindlimb syndactyly in homozygotes (*Mp/Mp*; Figure 1C). The eyelids in *Mp/Mp* animals never opened, however contrary to the original report, microphthalmia rather than anophthalmia was identified on dissection (Figure 1D). Further analyses revealed that the *Mp/Mp* limb phenotype ranged in severity from osseous fusions of the entire phalanges of digits 2–3–4 (Figure 1H), to simple soft tissue syndactyly affecting these digits (not shown). Digits 1 and 5 were never affected and no metatarsal abnormalities were identified. Consistent with the original report in which homozygotes were described as runted, we observed significant weight differences between mutant and Wt animals (Figure S1). *Mp* mutant phenotypes were fully penetrant throughout crosses of all genetic backgrounds tested (Figure S1).

### Ciliary body malformation and aberrant developmental Wnt signalling in the *Mp* eye

**Figure 2:**
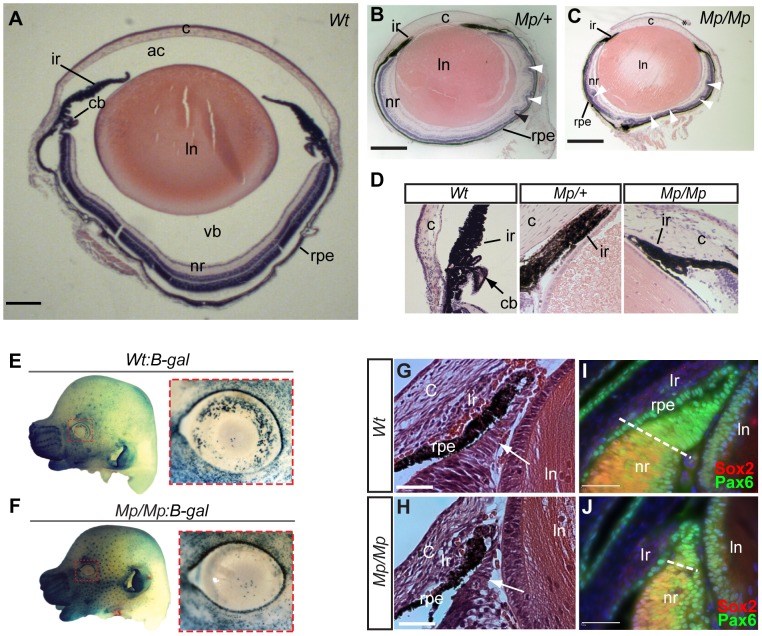
*Mp* eyes displayed structural defects and abnormal ciliary development. Histological comparison of eye tissues in Wt (A), *Mp/+*(B), and *Mp/Mp* (C) at P21 revealed severe pan-ocular defects. Mutant eyes displayed microphthalmia and retinal rosetting (arrowheads), together with loss of vitreous in the anterior chamber and between lens and retina. Lens size was also reduced in both mutant genotypes. Scale bars = 500 µm; sections are oriented in the sagittal plane. (D) Enlarged view of iris and the anterior region of retinas revealed the absence of ciliary body structures in both mutant types. (E–F) Genetic crosses of *Mp* with the Wnt-signalling reporter mouse line BAT-gal, revealed a significant reduction in galactosidase-positive cells in E14.5 *Mp* retinas compared to Wt, specifically in the dorsal and temporal regions of the anterior retina (*n = 8* per genotype). In contrast, non-ocular tissue displayed slightly increased staining in *Mp:B-gal* compared to Wt:*B-gal*, due to the slight increase in staining time in these samples. Ventral regions had low expression in both genotypes and were used as reference for quantitative comparison (Figure S2). (G–H) Histological analysis of anterior retinas at E15.5 revealed a reduction in non-pigmented ciliary body tissue (arrows) in *Mp/Mp* compared to Wt. (I–J) Immunohistochemical staining of anterior retinal structures with antibodies specific for Pax6 and Sox2 at E15.5 revealed reduction in the Pax6-positive and Sox2-negative non-pigmented ciliary epithelial region in *Mp*. Scale bars, 50 µm. The asterisk in C indicates artefactual disruption to the corneal epithelium during sample processing. Abbreviations: ac, anterior chamber; c, cornea; cb, ciliary body; ir, iris; ln, lens; nr, neural retina; rpe, retinal pigmented epithelium; vb, vitreous body.

Histological analysis at adult stages (P21) revealed pan-ocular structural defects in *Mp* (Figure 2A–C). In particular, the neural retina cell layers displayed severe rosetting and the vitreous was absent from the anterior chamber and posterior eye. Immunofluorescence studies revealed the nature of the retinal disruption with rosettes composed primarily of disorganised rod photoreceptors, and reduced numbers of cells at the inner nuclear layer (Figure S2). Furthermore, ectopic ganglion cells were identified in multiple regions of the *Mp/Mp* retina and expression of GFAP was observed, indicative of gross damage to the retina. In both *Mp/M* and *Mp/+*, the ciliary body was consistently absent however the iris appeared to form normally (Figure 2D). Although retinal lamination is not fully complete until approximately three weeks after birth, the ciliary body develops during mid gestation from non-pigmented ciliary epithelial cells. The anterior region of the early developing retina, and future ciliary epithelium, is marked specifically by canonical Wnt signalling and we therefore crossed *Mp* to BAT-gal [33], a Wnt-reporter strain that expresses beta-galactosidase in the presence of activated beta-catenin. In *Mp/Mp*-BAT-gal^+/−^ E14.5 mice, we detected significantly reduced canonical Wnt signalling in the anterior retina as shown by the loss of positively stained cells (Figure 2E–F, and Figure S2). Furthermore, at E15.5 a marked reduction in the size of the developing non-pigmented ciliary was observed upon histological staining as well as staining for the anterior retina marker Pax6 (Figure 2G–J). Furthermore, we revealed reduced total retinal size in *Mp/Mp* eyes at the same developmental stage (Figure S2). We therefore concluded that the *Mp* eye phenotype resulted from perturbations to normal ocular development, and first identifiable at the region of the developing ciliary epithelium.

### Genetic mapping of *Mp* to the *Fbn2* locus

**Figure 3:**
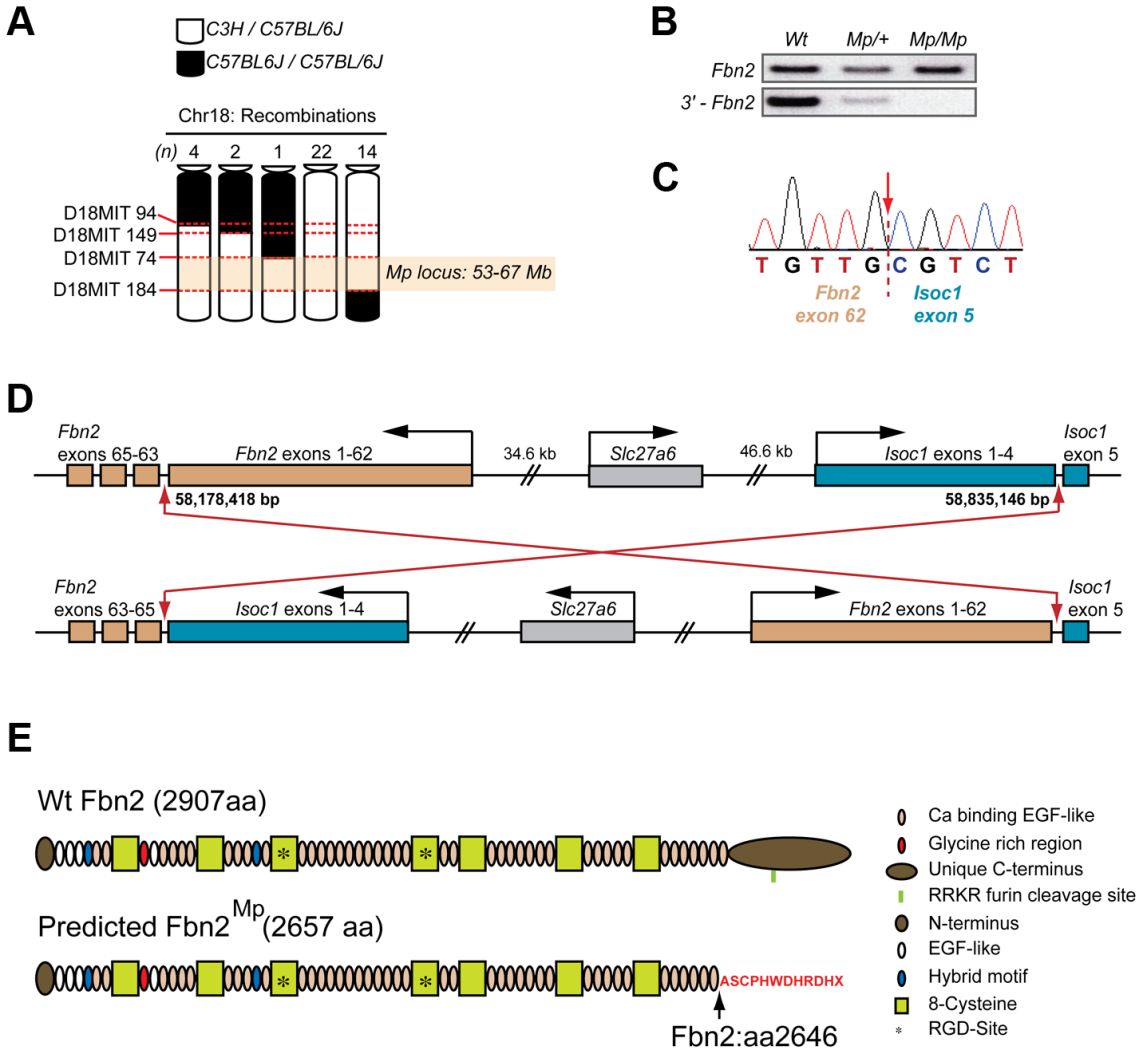
*Mp* mapped to a balanced 660 kb inversion on chromosome 18 disrupting *Fbn2* and *Isoc1*. (A) Haplotype analysis using DNA samples from 3^rd^ backcross (from parental strain C3H onto C57BL/6J) *Mp/+* animals (n = 43) for four microsatellite markers on chromosome 18 revealed there had been no meiotic recombination events between markers D18MIT74 and D18MIT184, defining an *Mp* candidate loci between 53–67 Mb (NCBI37/mm9 mouse assembly). (B) Semi-quantitative RT-PCR analysis of the *Fbn2* mRNA for each genotype revealed a failure to amplify the 3′-terminal region (bottom panel) of the transcript in *Mp/Mp* and an apparent reduction in *Mp/+*, compared to the normal amplification of a more central region of the *Fbn2* mRNA (top panel). (C) Sequence chromatogram from 3′-RACE revealed cDNA sequence from *Fbn2* exon 62 fused with sequence from *Isoc1*, a 5-exon gene positioned <1 Mb *in cis* from *Fbn2* on chromosome 18. *Fbn2* has 65 exons, *Isoc1* has 5 exons. The *Mp* transcript was composed of *Fbn2* exons 1–62 spliced directly to exon 5 of *Isoc1* with 100% homology and no extra nucleotides added or removed. (D) The *Mp* genomic rearrangement comprised a balanced inversion of chromosome 18 with breakpoints positioned in the 3′-introns of *Fbn2* (intron 62) and *Isoc1* (intron 4). Breakpoints were located at Chr18:58,178,418 and Chr18:58,835,246. Note that *Fbn2* is transcribed from the minus strand. (E) Fibrillin-2 Wt (top) and Fibrillin-2^Mp^ (bottom) proteins with domains indicated. Fibrillin-2^Mp^ is predicted to be missing the final calcium-binding EGF domain and its unique C-terminus, which have both been replaced by a sequence of 11 exogenous amino acids (red text), encoded out of frame from the *Isoc1* terminal exon and followed by stop codon. Note also the removal of the endogenous RKKR furin cleavage domain.

We performed a whole-genome scan using microsatellite markers on heterozygous *Mp* mice backcrossed to C57BL/6J, and identified enrichment for background strain C3H-specific alleles co-segregating with *Mp* at three consecutive markers on chromosome 18 (data not shown). During fine mapping, no recombination events between markers *D18Mit74* and *D18Mit184* were found in 43 *Mp/+* animals tested (Figure 3A). The *Mp* mutation was thus likely to be located between approximately 53 Mb and 67 Mb on chromosome 18. *Fbn2* (RefSeq Gene NM_010181), an extracellular matrix protein, maps within this interval. The hind-limb phenotype of *Mp* was consistent with previously published loss of function *Fbn2* mutations [30], [31], [32], [34]. Human mutations in *FBN2* are associated with crumpled ear helices in affected patients [35], [36], [37] and comparable ear phenotypes were observed in *Mp*. However, heterozygous *FBN2* mutations in humans do not cause ocular malformations and no ocular phenotype has been reported for Fibrillin-2 null mice [30], [31], [32], [34]. Furthermore, our histological examination of adult and neonatal Fbn2-null eyes revealed no ocular phenotype on genetic backgrounds analogous to *Mp* (Figure S3).

Nevertheless, we considered *Fbn2* to be a good candidate for *Mp*. Standard RT-PCR was unable to amplify a 3′- region of the *Fbn2* cDNA (Figure 3B). Using 3′-RACE we identified a fragment present in *Mp/Mp* but absent from Wt mRNA, and a corresponding absence of the Wt *Fbn2* amplicon in *Mp/Mp* (Figure S3). Cloning and sequence analysis of the *Mp*-specific *Fbn2* amplicon identified that *Fbn2^Mp^* was transcribed up to and including all of exon 62, following which the mRNA contained the full sequence of the terminal exon from *Isoc-1* (RefSeq ID NM_025478) (Figure 3C), a 5-exon gene of undetermined function located <1 Mb from *Fbn2* on chromosome 18. Using combinations of *Fbn2* and *Isoc1* specific primers we discovered that this fusion was reciprocal and balanced so that the terminal *Fbn2* exons (exons 63–65) replaced the final exon (exon 5) of the *Isoc1* transcript (Figure S3), creating a 7-exon mRNA. Sequencing genomic DNA confirmed this to be a balanced inversion of chromosome 18 [NCBI37/mm9: Chr18:58,178,418–58,835,146] (Figure 3D). The predicted consequence for the translated Fibrillin-2^Mp^ protein was replacement of the C-terminal 261 amino acids (aa) from position 2646, with an 11 aa out-of-frame extension followed by a termination codon, encoded by the out-of-frame final exon of *Isoc1* (Figure 3E). Fibrillin-2^Mp^ lacks the evolutionarily conserved predicted furin cleavage site present in the full-length protein (Fbn2 2769–73 aa; RRKR) [38].

In contrast, the inversion predicted nonsense-mediated decay of the *Isoc1^Mp^* transcript due to the frameshift-induced stop codon in the 5th of the 7 exons of this fusion gene [39], [40]. Consistent with this we observed approximately 50% reduction in *Isoc1^Mp^* mRNA levels by quantitative RT-PCR from developing eye tissue (Figure S3). The *Slc27a6* gene, encoding a member of the fatty-acid transport family protein, is located between *Fbn2* and *Isoc1* and its orientation was changed by virtue of its position at the centre of the *Mp* genomic rearrangement, possibly affecting its *cis*-regulation. However the gene itself was not structurally altered and we observed no change in the low level of *Slc27a6* expression that could be detected in Wt and *Mp* samples by RT-PCR (Figure S3). Additionally, signal was not detectable for *Slc27a6* or *Isoc1* at any stage when analysed by whole mount in situ hybridisation (WISH) in Wt and mutant samples (Figure S4). Although WISH is not sensitive enough provide conclusive proof, this data suggests that the developmental expression of these genes was not significantly changed or ectopically upregulated in *Mp* embryos. However, we could exclude loss of low-level expression of either of these genes as a cause of the ocular phenotype based on previous genetic studies. A chromosomal deletion causes the shaker-with-syndactylism (*sy*) phenotype extends at least between the microsatellite markers *D18Mit124* at 57.61 Mb (NCBI37/mm9 C57BL/6J) and *D18Mit205* at 58.95 Mb, encompassing *Isoc1* (58.82–58.84 Mb) and *Slc27a6* (58.72–58.77 Mb) as well as *Slc12a2* and *Fbn2* [34]. Mice heterozygous for *sy* have no morphological phenotype, and homozygotes have fused digits due to loss of *Fbn2* and are deaf because they lack *Slc12a2* [41]. The truncation of Fibrillin-2^Mp^ thus remained the sole candidate and we therefore focussed our investigations on the *Fbn2^Mp^* gene product.

### Fibrillin-2 distribution was disturbed in *Mp*

**Figure 4:**
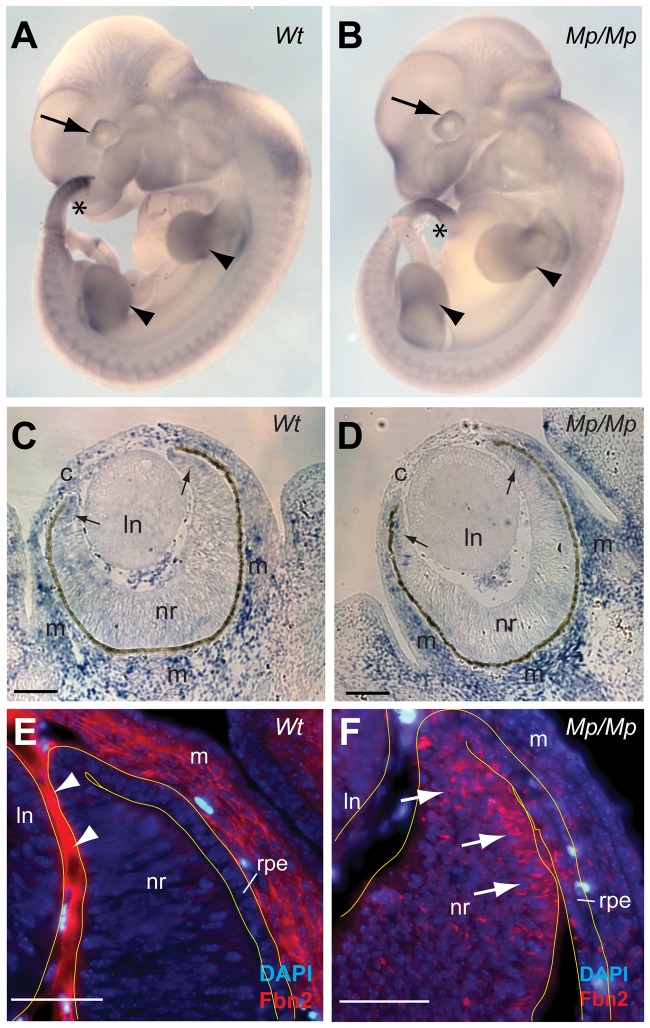
Fibrillin-2^Mp^ aggregated into large intracellular inclusions within the rough endoplasmic reticulum. (A–B) Whole Mount *In Situ Hybridisation* to *Fbn2* using antisense 3′-UTR riboprobes of *Fbn2* for Wt and *Isoc1* for *Mp/Mp* in early Wt and *Mp/Mp* embryos (E11.5) revealed the spaciotemporal expression of the variant *Fbn2* alleles appeared unaffected by the genomic inversion and that *cis*-regulation of the genes was unchanged. *Fbn2* was expressed in the developing eyes (arrows), limbs (arrowheads), and tail (asterisks), and expression was also observed in the somites. (C–D) Section *In Situ Hybridisation* for *Fbn2* at E13.5 again showed that mutant *Fbn2* expression was comparable to Wt in the eyes, with *Fbn2* identified in the periocular (m) and corneal (c) mesenchyme, and faintly in the anterior retina (arrows). No expression was identified in the lens (ln). Scale bars in, 100 µm. (E–F) Immunohistochemical analysis of the anterior region of E13.5 Wt eyes illustrated that Fibrillin-2 was localised to extracellular regions in the corneal mesenchyme and in the region of apposition between the lens and neural retina (arrowheads). In contrast, Fibrillin-2^Mp^ in *Mp/Mp* eyes was not observed in these extracellular locations but instead appeared to be retained within cells throughout the developing eye, with the anterior neural retina (arrows) and adjacent RPE displaying the most numbers of positive-cells. Scale bars, 50 µm.

We performed whole-mount and tissue section *in situ* hybridisation to determine the embryonic expression patterns of *Fbn2* Wt and *Mp* mouse embryos. *Fbn2* showed site and stage specific expression in the developing eye, limb and tail in early embryos and gene expression patterns did not differ between Wt and *Mp* in any tissue or at stage analysed (Figure 4A & B; and Figure S4). Expression of *Fbn2* has recently been well documented in the developing mouse eye, with strong expression observed in the developing non-pigmented ciliary epithelium at the anterior retina, with expression also observed in cells of the cornea and the periocular mesenchyme [42]. Similarly, we observed *Fbn2* expression in our analysis of Wt and *Mp/Mp* eyes at E13.5, with no differences observed between genotypes (Figure 4C–D). Consistent with these sites of expression, using the Fibrillin-2 specific polyclonal pAb868 antibody, we observed Wt Fibrillin-2 protein in extracellular regions of the periocular mesenchyme and in the anterior region of the eye between the neural retina and lens (Figure 4E). In contrast, Fibrillin-2^Mp^ was absent from these extracellular areas and instead was observed within cells of the anterior eye (Figure 4F), with the strongest signal detected in the non-pigmented ciliary region. Fibrillin-2^Mp^ was also observed in the retinal pigmented epithelium (RPE) and in the corneal mesenchyme (consistent with *Fbn2* mRNA distribution). We then looked at Fibrillin-2 distribution in other tissues known to express *Fbn2* and revealed similarly marked differences in protein localisation between Wt and *Mp* (Figure S4). In all samples analysed (mouse embryonic fibroblasts (MEFs); femoral articular cartilage and skin), Wt Fibrillin-2 protein was detectable in typical extracellular microfibril structures, but mutant protein was confined to intracellular foci adjacent to cell nuclei and was not observed in extracellular regions.

### Fibrillin-2^Mp^ formed stable fibrils within the endoplasmic reticulum

**Figure 5:**
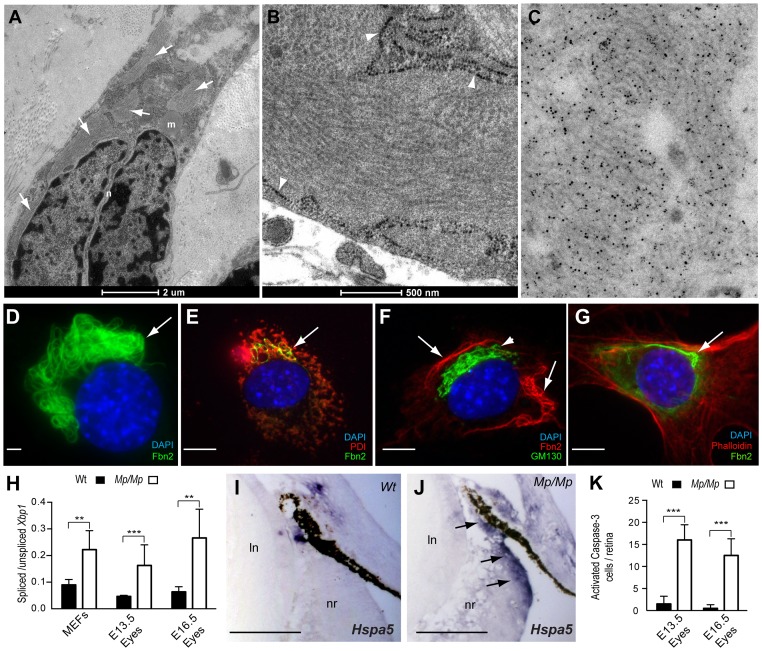
Fibrillin-2^Mp^ inclusions resulted in ER-stress and cell death in the developing *Mp* eye. (A) TEM micrograph of *Mp/Mp* ocular scleral cells revealed multiple intracellular inclusions located within the enlarged rough endoplasmic reticulum (rER) membrane (arrows). Scale bar, 2 µm. (B) Enlarged micrograph illustrating the structural periodicity of the inclusions with regular banding, possibly representing heterotypic fibrils. Arrowheads indicate rER membrane. Scale bar, 500 nm. (C) Immuno-gold labelling of the inclusions in *Mp/Mp* scleral cells with polyclonal anti-Fibrillin-2 antibody pAb868 and beads conjugated to secondary antibody revealed the inclusions to be composed of Fibrillin-2. (D) Fibrillin-2 immunofluorescence in *Mp* MEF cultures illustrated the perinuclear localisation and bundle-like organisation of the mutant protein aggregates. Scale bar, 10 µm. (E) Co-immunofluorescence with anti-ER marker PDI (red) confirmed the Fibrillin-2^Mp^ (green) inclusions colocalised within the ER (yellow stain; arrow). (F) Staining with the golgi-specific marker GM130 (green staining; arrowhead) showed that Fibrillin-2^Mp^ (red staining, arrows) was not localised within the Golgi. (G) Non-overlapping staining of Fibrillin-2 (green) and phalloidin (red) indicated that the inclusions were not located in the cytoplasm. Scale bars in E–G, 100 µm. (H) The ratio of *Xbp-1s* to *Xbp-1u* was significantly increased in *Mp/Mp* compared to Wt in RNA samples collected from MEFs and from *Mp/Mp* eyes at E13.5 and E16.5. (I) Section *In Situ* for ER-stress marker *Hspa5* mRNA revealed no staining in Wt retinas at E16.5, (J) however there was clear signal in the non-pigmented ciliary epithelium (arrows) in *Mp/Mp*. Scale bars, 50 µm. (K) (J) Activated-Caspase-3 stained cells from E13.5 and E16.5 Wt and *Mp/Mp* retinas were quantified and revealed significant increase in mutant eyes. Error bars are s.d. for H & K (**P<0.005; *** *P*<0.001).

To further identify the consequence of the *Mp* truncation to Fibrillin-2 distribution, and to explore possible consequences to expressing cells, we performed transmission electron microscopy (TEM) of eye scleral cells on mutant samples. These revealed inclusions located within the enlarged rough endoplasmic reticulum (rER) lumen that were organised into thick fibril-like aggregates, with apparent structural periodicity (Figure 5A–B). The inclusions were confirmed as Fibrillin-2 positive by immuno-EM (Figure 5C) and by immunofluorescence (Figure 5D). Deposits of Fibrillin-1-containing fibrils with similar ultrastructural appearance to the Fibrillin-2^Mp^ inclusions have been previously identified in extracellular normal human cartilage matrix [43], however the inclusions in our study did not contain Fibrillin-1 when tested by immunofluorescence (Figure S4), or by immuno-EM (not shown), and were exclusively intracellular. Nevertheless, both these results suggest that under some physiological conditions, Fibrillins can produce atypically banded fibrils. Immunofluorescence confirmed that the Fibrillin-2^Mp^ inclusions were ER-specific, and did not localise within the golgi (figure 5E–G).

### Fibrillin-2^Mp^ inclusions triggered ER-stress

We hypothesised that the presence of large inclusions could disrupt normal ER mechanisms and secretory function. First, we analysed expression of *Xbp1*, a widely used marker ER-stress or the unfolded protein response (UPR)[44]. Specifically, a quantitative assay was devised to measure the ratios of unspliced versus UPR-specific *Xbp1* transcripts. Validation was performed with cells cultured with increasing concentrations of tunicamycin (not shown). We then assayed *Mp/Mp* and Wt RNA samples from MEFs and from dissected E13.5 and E16.5 embryonic eye tissues, and found that relative levels of *Xbp1s* were significantly increased in all *Mp/Mp* samples compared to Wt (Figure 5H). We next analysed embryonic ocular expression of the UPR chaperone protein *Hspa5*, and observed expression in *Mp*, but not in Wt, specifically in the ciliary epithelium (Figure 5I–J). Immunostaining for Protein Disulphide Isomerase (PDI), revealed that *Mp* embryos had enhanced staining in the non-pigmented ciliary epithelium and adjoining RPE, co-localising with the Fibrillin-2^Mp^ inclusion-positive cells, whereas equivalent Wt cells were negative for PDI expression (Figure S5). In combination, these data confirmed ER-stress in developing *Mp* eyes, in regions consistent with the distribution of Fibrillin-2^Mp^ inclusions at the anterior developing ciliary margin.

We then investigated whether the presence of Fibrillin-2^Mp^ inclusions resulted in an increase in apoptosis in the developing *Mp* eye. We reasoned this could provide a straightforward explanation for the loss of retinal and ciliary epithelial cells observed in *Mp*. Immunostaining for activated Caspase-3 revealed increased numbers of apoptotic cells in the embryonic mutant retinas compared to Wt (Figure 5K). The positive cells were evenly dispersed throughout the entire neural retina, but surprisingly these were not enriched in the areas with the highest number of Fibrillin-2^Mp^-positive cells, i.e. the non-pigmented ciliary epithelium and adjacent RPE (Figure S5). This suggested that additional or alternative pathological mechanisms account for the disruptions to the developing anterior eye in *Mp*.

### Protein secretion defects in UPR-affected cells

**Figure 6:**
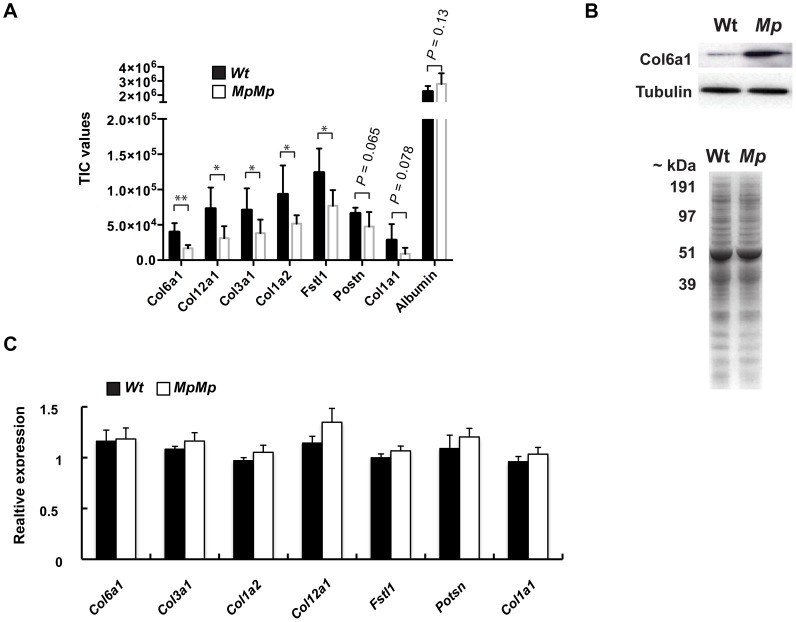
Fbn2^Mp^ inclusions affect the secretion of extracellular proteins. (A) Conditioned media samples from primary MEF cultures collected from independent embryos (*n = 5* cell lines for each genotype) revealed reductions in secretion of five separate Collagen-family proteins, Periostin and Follistatin-like 1 by quantitative proteomics using comparative peptide TIC analysis. As a control, the detected levels of BSA supplemented into the culture media were equivalent (Plots: mean TIC values of proteins secreted from *Mp/Mp* and Wt cells; error bars, s.d. *P<0.05; **P<0.01). (B) *Top:* Immunoblot of total MEF cell lysates with anti-Col6a1 antibody revealed increased intracellular protein in *Mp/Mp* versus Wt. *Below:* Coomassie stained SDS-PAGE gel of conditioned media showed no observable total protein differences between samples. (C) Quantitative RT-PCR analysis of RNA from MEF samples revealed no significant reduction in mRNA levels of these genes between the two genotypes, indicating that transcription was not the primary cause of reduced secretion. RNA was extracted from the same cultures as the conditioned media was collected. Graph represents the mean cT values for Wt and *Mp/Mp*, divided by the respective mean cT values for *Actin* mRNA (All PCR reactions were carried out in triplicate, *n = 5* per genotype, error bars, s.d. No differences between means reached significance, Students t-test).

We then considered whether the presence of Fibrillin-2^Mp^ inclusions and the consequential induction of UPR prevented the secretion of other extra-cellular proteins expressed in the same cells. We took multiple independent primary Wt and *Mp* MEF cultures and analysed their conditioned media using quantitative label-free mass-spectrometry. We found reduction in the secretion of many proteins, of which the most severely affected were collagens (Col6a1; Col3a1; Col1a2; Col1a1; and Col12a1), Periostin and Follistatin-like 1 (Figure 6A). Confirmation of the MS data was performed by immunoblotting for one of the identified enriched proteins, Col6a1, which revealed elevated intracellular protein levels in *Mp* (Figure 6B). Albumin supplemented into the culture media was detected at equivalent levels by these analyses, and Coomassie-stained SDS-PAGE gels of total secreted proteins were used as a further experimental control. To ensure that transcriptional down-regulation was not responsible for the differences seen in protein levels, we assessed the relative mRNA levels of the affected proteins and found the expression of all the genes to be equivalent to Wt (Figure 6C). As a complementary assay, we transfected Wt human RPE1 cells with a plasmid encoding a tagged secreted protein (Wnt3A-FLAG) and added tunicamycin (Tn) to the cell culture medium to induce the UPR. Increased levels of Hspa5 in cell lysates confirmed that the Tn-treated cells were undergoing the UPR, and we saw a coincident reduction to secreted Wnt-FLAG in these cells compared to similarly transfected cells without Tn (Figure S6). We also observed a reduction in the mass of the migrating Wnt-FLAG protein, consistent with reduced glycosylation in the Tn-treated cells. Thus, Fibrillin-2^Mp^ inclusions have a negative effect on protein secretion, and this is a likely consequence of ER-stress.

## Discussion

In humans, mutations in the *FIBRILLIN* gene family result in fibrillinopathies, diseases that display a broad phenotypic spectrum consistent with their developmental expression patterns [45], [46]. Autosomal dominant (AD), and less commonly autosomal recessive, mutations in *FBN1* result in the skeletal, cardiovascular and ocular features of Marfan syndrome (MIM #154700), whereas AD *FBN2* mutations typically affect skeletal regions in arthrogryposis, in which patients usually present with long digits, distal joint contractures and crumpled ears, but rarely with ocular or cardiovascular complications. Haploinsufficiency for *FBN1* appears to be the mechanism responsible for the majority of cases of Marfan syndrome [47]. However, dominant-negative effects appear to be responsible for the very severe phenotypes associated with neonatal and infantile Marfan syndrome. In these cases the causative mutations cluster within exons 24–32 [48]. The cognate region of *FBN2* is the most commonly mutated in Beal syndrome and would plausibly be associated with a similar dominant negative effect [49]. An interesting phenotype of neonatal progeroid features, Marfan syndrome and generalised lipodytrophy (Wiedemann-Rautenstrauch syndrome) has recently been described in association with *FBN1* mutations located in the C-terminal domain of the protein [50], [51], [52]. The pathogenic mechanism is not yet clear but it will be important to determine if the mutant protein is normally secreted.

*Mp* displayed phenotypic overlap with the human fibrillinopathies, including contractures, small ears and skeletal malformations in the limbs. Studies in mice have shown that the *Fibrillins* are differentially expressed through embryonic development, with *Fbn2* transcripts appearing first and coinciding with early morphogenesis and elastic fibre assembly. The overlapping, but delayed expression of *Fbn1* is consistent with a more structural role after defined organ structures have been established. Although both Fibrillin-1 and Fibrillin-2 are present during embryogenesis, neither is absolutely necessary, as microfibrils and elastic fibres are formed in the absence of either protein. To date, five separate loss of function mouse mutations in *Fbn2* have been described, all with highly penetrant homozygous hind-limb syndactyly but no ocular phenotype [30], [31], [32]. Thus *Mp* is likely a Fibrillin-2 null mutation in limb development but its ocular phenotype clearly resulted from a separate mechanism, specific to its genomic inversion.

It cannot be ruled out that the extraneous amino acids added by the out-of-frame fusion to *Isoc1* conferred a gain-of-function to this chimeric protein, preventing its secretion. Although no pro-protein cleavage by furin has previously been shown directly for Fibrillin-2 at the C-terminal domain, the inclusion data presented here, together with the high degree of conservation of furin-cleavage motifs among all Fibrillins [53], strongly suggests that this processing is common to all Fibrillins but is lost in Fibrillin-2^Mp^. It seems likely then that the failure of proprotein processing is the cause of the intracellular accumulations, and it is the failure of secretion of Fibrillin-2^Mp^ that is the cause of the UPR.

In addition to the primary secretion failure, ER-stress is likely to reduce the protein synthesis and/or secretory capacity of affected cells [54]. We observed a significant reduction in several secreted proteins from *Mp* cultured primary cells. The cells in the developing *Mp* eye that contain the highest number of Fibrillin-2^Mp^ inclusions and display a UPR are those at the anterior rim of the optic cup in the non-pigmented ciliary epithelium. These cells produce various secreted factors including collagen proteins required for the accumulation and maintenance of ocular vitreous [55], [56]. In this context it is interesting that the quantitative MS analysis of the *Mp* versus Wt MEFs identified several collagen molecules among the most altered of the secreted proteins. The reduction of posterior vitreous chamber is an early and prominent feature of the developmental pathology in the *Mp* eye. While this feature could be due to failure of collagen secretion, is it also plausibly the consequence of a failure in morphogenesis of the ciliary apparatus itself. Cells in the *Fbn2*-expressing region of the embryonic eye also secrete Wnt2b, which is of interest in the context of the reduction to BAT-gal reporter activity that we observed in *Mp* in this region. Disruption of Wnt2b production results in pan-ocular phenotypes overlapping with those observed in *Mp*, including retinal rosettes, lens abnormalities, iris hypoplasia and the absence of vitreous [27], [57]. Wnt2b from the anterior retina has also been shown to confer layer-organising properties to the central retina [58]. In *Mp*, a reduction of Wnt2b from the ciliary body may be predicted as a result of UPR-mediated apoptosis (i.e. fewer cells to produce), but may also result from reduction to the normal secretion of Wnt2b through the ER of Fbn2 expressing cells, thus reducing the amount of bioactive paracrine signal available to the developing eye.

One question that arises from this study is why are developing eyes the only tissues that are sensitive to Fibrillin-2^Mp^ ER-stress, and not other expressing cells? If we trust the phenotype as an assay both in the Fbn2 null and *Mp* animals, this would suggest that Fibrillin-2 has few non- redundant roles (digit development) and perhaps in *Mp* only the developing retinal cells that normally secrete Fbn2 are co-expressing gene products that are also critical for development. Skin tissue appeared surprisingly lax compared to Wt (data not shown), and animals were runted. These data suggest there may also be pathogenic consequences to other tissues, but that these do not manifest as profoundly as in the developing eyes, indicating either elevated sensitivity to ER-stress, or intolerance to reductions of co-secreted proteins in the developing eye.

It is possible that there are other mechanisms mediating the protein secretion abnormalities observed in *Mp* in addition to the ER stress. One such mechanism could relate to the capacity for the ER-localised Fibrillin-2^Mp^ to bind other proteins. Mutations in *COMP* cause human pseudoachondroplasia (PSACH; MIM#177170) and multiple epiphyseal dysplasia (EDM1; MIM#132400), diseases of short stature and osteoarthiritis, both of which are associated with retention of mutant COMP within the rER. The mutations cause a variety of plausibly pathological effects including: a decrease in cellular viability, failure of COMP to be secreted into the extracellular matrix, and a *trans*-acting effect on co-expressed proteins [59], [60], [61]. Mutant COMP accumulates within the enlarged rER of *in vivo* cartilage and associates with co-expressed type IX collagen, with the secretion and extracellular localisation of these factors correspondingly affected. However, recent analyses of PSACH mouse models have shown that these effects are not mediated *via* UPR but rather through a novel form of chondrocyte stress [62]. The best-known endogenous Fibrillin-2 binding partners are the family of latent TGFbeta binding proteins (LTBPs). In this regard, the observed reduction in secretion of the modulator of BMP signalling factor, Follistatin-like 1, may be interesting, although there is currently no direct evidence that this protein binds to either LTBPs or Fibrillin-2. Interestingly Fibrillin-1 is also a binding partner of Fibrillin-2 and is similarly processed through the ER, but we were not able to find any evidence of Fibrillin-1 colocalisation to the *Mp* inclusions (see Figure S4). However, we did observe increased intracellular retention of Col6a1, suggesting that intracellular association of Collagens to Fibrillin-2^Mp^ may occur. Future work will be required to determine whether collagens, or Wnt2b, directly associate with Fibrillin-2^Mp^ inclusions in the developing eye.

We have shown here that the ocular phenotypes in *Mp* are caused by a truncating mutation in *Fbn2*. Perturbation of normal ER function, via mutations similar to *Mp* in proteins that functionally pass through the ER, may lead to abnormal phenotypes that cannot be predicted from knowledge of the primary mutant allele. The terms antimorphic, and the more commonly used dominant-negative, are used to describe a worse than heterozygous effect on the function of the protein encoded by the mutated gene or on the cognate functional pathway. Here we propose that the *Fbn2^Mp^* mutation is disrupting one or more developmental functions in which the gene product itself plays no direct part. The compromised gene products are simply expressed in the same cell and share a secretory pathway. We suggest the term *synodiporic* (fellow traveller) effect for this phenomenon. Thus, the *Mp* mouse provides clues towards a genetic mechanism of activating disease mutation, which may be phenotypically more severe than those predicted simply by loss of protein function, or may elicit an entirely novel phenotype due to ER perturbation or any other mechanism affecting the production or availability of secondary co-expressed proteins. This has wide-ranging consequences for disease-gene mapping, and for disease management and therapy in human conditions.

## Materials and Methods

### Ethics statement

The *Mp* mouse was re-derived from frozen sperm obtained from the Jackson Lab (http://www.jax.org/mmdb/results/Sp_Mp.htmlx). All work was carried out under UK Home Office Project License 60/3785 (IJ Jackson, MRC Human Genetics Unit).

### Rederivation and phenotyping of *Mp*

Heterozygote (*Mp/+*) sperm was used to perform intra-cytoplasmic sperm injection using oocytes from a donor female F1 (CBA x C57BL/6J). Phenotypically affected offspring were crossed to C57BL/6J to facilitate genetic mapping and to CD1 for phenotype and penetrance analysis. The *Mp* mutation phenotype has been described [29]. Heterozygote animals were identified by small eyes with/or without reduced pinnae size. Apparent anophthalmia (demonstrated by failure to open eyelids) in combination with distal limb syndactyly was used to identify homozygote animals. Records of each phenotype were accumulated for penetrance analysis in crosses to both C57BL/6J and CD1 strains.

### Skeletal analysis

Adult animals were culled with lethal intravenous doses of Euthatal (Merial Animal Health Ltd. Essex, UK), treated in 95% ethanol for 24 hours; then 72 hours in 100% acetone; a minimum of 3 days in stain solution (1 part 0.3% alcian blue in 70% ethanol; 1 part 0.1% alazarin red in 95% ethanol; and 1 part acetic acid, in 17 parts 70% ethanol); 3 days in 1% potassium hydroxide (KOH); 3 days in 1% KOH/30% glycerol; and 24 hours in each 1% KOH/50% glycerol and 1% KOH/70% glycerol and finally stored in 100% glycerol.

### Mouse eye processing for histology and immunofluorescence

Embryos and eyes were dissected and rinsed in cold phosphate buffered saline (PBS) and then fixed in 4% PFA overnight at 4°C; rinsed again in PBS and dehydrated in serial dilutions of ethanol and paraffin embedded. All adult eye sections were cut using a standard microtome at 12 µm and oriented in the sagittal plane; embryonic sections were cut at 6 µm and oriented in the coronal plane. Haemotoxylin and Eosin staining was performed according to standard methods.

### qRT-PCR and PCR

Details of all primers and sequences used in this study are in Table S1. DNA was extracted according to established protocols. RNA was isolated from tissues or from cultured mouse embryonic fibroblast (MEF) cells using TRIzol Reagent (Invitrogen) according to the manufacturers instructions. For cDNA, 1^st^ Strand cDNA Synthesis Kit for RT-PCR (Roche), or Superscript III (Invitrogen) were used according to the manufacturers instructions. All PCR reactions were carried out with primers at 0.2 µM. Cycle conditions: 95°C×3 min; followed by 30 cycles of 95°C×30 s; 58°C×45 s; 72°C×45 s; followed by 10 min at 72°C. Quantitative RT-PCR was performed as above using Brilliant II SYBR Green QPCR Master Mix (Agilent) and an ABI HT7900.

### Genome-wide mapping of *Mp*

Microsatellite markers were used for PCR-based mapping using genomic DNA samples from heterozygous (*n* = 38) offspring from the 3^rd^ generation of outcrosses to C57BL/6J (for Wt, *n* = 30). In total 73× markers were selected that were informative between C3H and C57BL/6J alleles. A list of the markers used and peak sizes for each allele are available in Table S1. Equimolar samples were pooled together as either wild type or heterozygote DNA. Amplicons were analysed on the ABI PRISM 310 Genetic Analyzer. Peak heights for each allele were recorded and the ratios of C57BL/6J:C3H were calculated. For the chromosome 18 locus, non-fluorescently labeled microsatellite PCR products were resolved on 4% agarose gels. Oligonucleotides for fine mapping chromosome 18 are listed in Table S1.

### 3′-End cDNA amplification

Rapid Amplification of cDNA Ends (RACE) was used for the identification of the 3′-end of the *Fbn2* transcript using an adaptor oligonucleotide primer (5′-GACTCGAGTCGACATCGATTTTTTTTTTTTTTTTT-3′) as the only primer in separate reverse transcription reactions with RNA extracted from phenotypically mutant and Wt animals as described above, using 1^st^ Strand cDNA Synthesis Kit for RT-PCR (Roche) with standard conditions. The single-stranded cDNA species (cDNA) were then used in gene-specific PCR reactions with an adaptor-only primer (5′-GACTCGAGTCGACATCG-3′) and the *Fbn2*-specific primer (5′-GTCTCAGCCTTCCCTCTGTG-3′). Products from these separate reactions were then gel electrophoresed and candidate bands were excised and TA-cloned into the pGEM-Easy vector system (Promega), according to the manufacturers instructions. Sequence analysis was performed using T7 and SP6 vector-specific primers and Sequencher Version 4.8 (Gene Codes Corp. MI, USA) and then used as query sequences in BLAT search alignments against the mouse genome (http://genome.ucsc.edu/cgi-bin/hgBlat). A PCR-based genotyping assay was devised based on the genomic rearrangement identified at the *Mp* locus.

### Transmission-Electron Microscopy (TEM)

Tissues were collected from freshly culled neonates (P2 and P8) and placed into sterile serum-free DMEM (Gibco). For ultrastructural observation, the tissue was fixed for 1–3 hours in 1.5% Glutaraldehyde/1.5% Paraformaldehyde with 0.05% tannic acid in DMEM, rinsed in DMEM then immersed in 1% OsO_4_ in DMEM for one hour. Tissues were dehydrated in a graded series of ethanol to 100%, washed in propylene oxide, then infiltrated and embedded in Spurr's epoxy. 60–90 nm ultrathin sections were contrasted with saturated uranyl acetate in 50% ethanol for 15 minutes followed by lead citrate for 3 minutes. For immunocytochemistry, fresh tissue was fixed in 0.1% glutaraldehyde/4% paraformaldehyde for 30 minutes on ice, rinsed in DMEM then 0.15 M TRIS-HCl (pH 7.4) overnight. Tissues were then exposed to a graded series of ethanol dilutions to 90% at progressively lower temperature to −20°C, infiltrated in LR White embedding media at −20°C, then polymerized at 60°C in a nitrogen atmosphere. Ultrathin sections mounted on formvar coated 1×2 mm nickel slot grids were immunolabeled using antibody pAb0868 specific to Fbn2, pAb9543 specific to Fbn1 and also control antibodies of irrelevant specificity followed by a combination of −5 and −10 nm secondary gold conjugates as previously described [42]. Sections were examined on either a Philips EM410LS TEM or an FEI G20 TEM operated at 120 kV.

### Primary MEF cultures

Genotyped embryos were collected at E13.5 and the limbs and skin from each embryo were then finely chopped and cultured in DMEM (Gibco) with 20% foetal calf serum and 1% penicillin/streptomycin at 37°C; 5% CO_2_; 3% O_2_. The Wnt-FLAG containing plasmid was a kind gift from Dr Angela Lee and modified from a PCMxGFP2FLAG vector. Transfection of RPE1 cells with Wnt-FLAG were performed using Lipofectamine-LTX (Invitrogen), with pcDNA3.1 containing enhanced-GFP to control for transfection efficiency. Tunicamycin (Sigma) was used at a concentration of 0.5 µg/ml and carried in DMSO. Non-Tn treated cells were exposed to carrier alone.

### *Xbp1* splicing assay

Alternatively spliced *Xbp1*transcripts were differentially identified using RT-PCR and the ABI PRISM 310 Genetic Analyzer, using a FAM-labeled forward, and an unlabelled reverse primers specific for the spliced region of *Xbp1* (Table S1). RNA samples were collected from embryonic eyes and MEF cultures and cDNA was synthesized. The unspliced (*Xbp1u*) transcript was 289 bp and the spliced transcript (*Xbp1s*) was 263 bp. The assay was verified using tunicamycin to induce ER-stress in cultured MEFs (data not shown). Peak heights for each amplicon were measured using the ABI PRISM 310 Genetic Analyzer, and means were calculated and displayed graphically.

### Immunohistochemistry

All antibodies used in this study are listed in Table S2. For immunofluorescence, primary MEFs were cultured on glass coverslips as described above for 72 hours and media was replaced with serum-free media for 16 hours. Coverslips were fixed (i) for PDI and GM130 staining- with 3.7% formaldehyde on ice for 10 minutes, or (ii) for Fbn2 with acetone at −20°C for 15 minutes, rinsed once in PBS and then permeabilised in PBS+0.5% Triton-X-100 (Sigma) for 10 minutes, blocked in PBS+10% serum for 1 hour, and incubated with primary antibodies overnight at 4°C. Coverslips were washed 3×5 minutes in PBS, and incubated in Alexa Fluor F(ab′) fragment (Invitrogen) secondary antibodies diluted 1∶1000 in blocking buffer for 1 hour and mounted with ProLong Gold Antifade reagent (Invitrogen). For immunohistochemistry, paraffin sections were de-waxed and antigen retrieval was performed by boiling slides in 0.1 M Citrate buffer (pH 6.0) for 5 minutes. Paraffin or cryosection samples were then rinsed in PBS and blocked for 1 hr in buffer containing 10% serum (heat inactivated at 60°C for 1 hr); 1% BSA; 0.1% Tween-20; 0.05% Triton-X-100, all in PBS. Primary and secondary antibodies were applied as before. Colorimetric immunohistochemistry was performed using Vectastain ABC kits (Vector Laboratories) with alkaline phosphatase detection and BM Purple (Roche). Imaging was performed using a Zeiss Axioplan II fluorescence microscope with Plan-neofluar objectives. Images were captured using a Coolsnap HQ CCD camera (Photometrics Ltd, Tucson, AZ). Image analysis was performed using IPLab Spectrum Software (Scanalytics Corp, Fairfax, VA).

### RNA In Situ analysis and riboprobe synthesis

Riboprobe synthesis primers are listed in Table S1. Purified PCR products were incubated for 37°C for 2 h in a 20 µl reaction containing: 2 µl transcription buffer; 1 µl RNase inhibitor; 2 µl DIG RNA labelling mix and T7 RNA polymerase (all Roche); in ultra-pure H_2_O. Riboprobes were then DNAseI treated and Sodium-acetate/Ethanol precipitated. Whole-mount in situ hybridization to mouse embryos was carried out as previously described [63]. For section *in situ*, tissue sections were prepared as described for immunohistochemistry, and processed as described in J. Rainger's doctoral thesis (available on request). X-gal staining was performed on dissected embryos for beta-galactosidase activity as described in [18].

### Secretion assays

For secretion assays, 1×10^5^ cells were seeded into 6-well plates and cultured under normal conditions for 3 days. Five independent cell lines were used per genotype. Media was removed and replaced with 1 ml DMEM (Gibco) with reduced serum and cultured for a further 72 hours. The conditioned media was collected and 500 µl was concentrated 10 fold using an Amicon Ultra 30 K (Millipore) centrifugal filter according to the manufacturers instructions. Concentrated conditioned media samples were then both (i) run on 10% NuPAGE Novex Bis-Tris Mini Gel SDS polyacrylamide gel (Invitrogen) and stained with 0.05% Coomassie Brilliant Blue (40% methanol, 10% acetic acid), and (ii) measured for total protein by Bradford assay, to ensure equal protein content per sample. Their protein composition in these media were analysed using a label-free shotgun proteomics method according to a previous report [64] with some modifications. First, the database search was performed using Proteome Discover (version 1.2) or Peaks (Peaks Scientific, version 6.0) with peptide false discovery rate constrained at 1%. Second, parent ion mass tolerance and fragment ion mass tolerances were set at 5 ppm and 0.6 Da. Third, Sieve software (version 2.0; Thermo Scientific) and Peaks (Peaks Scientific) were used to compare protein abundance with a total ion current normalization. Only more than two peptides per protein detectable was considered and each protein was expressed as a ratio *Mp/Mp*:Wt according to Asara et al [65]. For transcript analyses, cells were dissociated and counted using a haemocytometer and total RNA extractions were performed with Trizol reagent (Invitrogen) and cDNA prepared. qRT-PCR was performed on ABI HT7900 using 0.5 M sequence-specific primers for *Col6a1*; *Col3a1*; *Col1a2*; *Col12a1*; *Fstl1*; *Postn*; and *Actn1* with Brilliant II SYBR Green QPCR mix (Agilent) and 0.4 µl of cDNA template per 20 µl reaction. Each sample was run in triplicate per qRT-PCR reaction and the data presented represents the mean of 5 independent samples per genotype and three separate qRT-PCR technical repeat. For relative expression, all CT values were normalized to CT-*ß-Actin*.

### Immunoblotting

Protein samples were run on NuPage SDS polyacrylamide gels and transferred to a PVDF membrane, blocked for 1 hour in 5% non-fat dry milk diluted in PBS and incubated overnight in primary antibodies diluted in block. Membranes were washed extensively in PBS and incubated in the relevant secondary IgG-HRP antibody diluted in block for 1 hour at room temperature. Membranes were then washed again and immuno-complexes were visualized with Amersham ECL Western Blotting Detection Reagent (GE Healthcare Life Sciences).

## Supporting Information

Figure S1 (A) Average weights (in grams) recorded between postnatal days 4 and P21 showed reduced weight of *Mp/Mp* from P4 compared to Wt and *Mp/+.* In addition, both mutant types failed to gain weight compared to Wt. Error bars, s.d. (B) Phenotype data from out-crosses (top) and intercrosses (bottom) to C57BL6/J and CD1 genetic backgrounds revealed that the *Mp* phenotype was fully penetrant on both strains.
(TIF)

Figure S2 (A) Haemotoxylin and eosin staining of P21 retinal tissue revealed retinal rosetting affecting the inner nuclear layer cells (arrows). Scale bar, 100 µm. (B) Immunohistochemical marker analysis revealed gross disruption to *Mp* retinas at P21. Staining for Rhodopsin was localised to punctate regions of the mutant retina (*arrows*), consistent with rosette foci, and was not identified in the outer segment of the retina, in contrast to the Wt retina where Rhodopsin signal was localised to the outer segment (arrows) and the outer nuclear layer (arrowheads). DAPI counterstaining illustrated the disruption to the normal lamination of the *Mp* retina compared to Wt. Vsx2 antibody staining displayed a reduced number of cells in the *Mp* inner nuclear layer, and staining for Calbindin was reduced in the *Mp* outer plexiform layer (arrows), indicating a disruption to the projections emanating from horizontal cells of this region of the retina. GFAP (glial fibrillary acidic protein) staining, a marker for Muller glial cells and retinal astrocytes but also retinal stress responses, was markedly increased in mutant eyes. Brn3a, expressed in retinal ganglion cells (arrows) displayed ectopic staining in multiple regions of the *Mp/Mp* retina (arrowheads) and revealed a reduction to the typical ganglion layer compared to Wt. Abbreviations: gcl, ganglion cell layer; inl, inner nuclear layer; ipl, inner plexiform layer; is, inner segment; onl, outer nuclear layer; opl, outer plexiform layer; os, outer segment; rpe, retinal pigmented epithelium. Scale bar = 100 µm. (C) Mean retinal widths at E15.5 measured in voxel units using OPT data from whole embryonic heads. Error bars, s.d. Wt *n* = 5; *Mp/Mp n* = 8. ***P<0.001 (Student's *t-*test). (D) Quantitative ß-galactosidase staining of *Wt:BAT-Gal* and *MpMp:BAT-Gal* eyes at E14.5 was performed by the segmentation of the eye into 6 regions of interest (ROI) and staining intensity was measured for each ROI using Photoshop software (Adobe) and normalised to the ventral ROI for each eye analysed. *n = *8 eyes per genotype. The mean of each region was used to graph the difference between genotypes for each comparative ROI. Error bars, 95% confidence intervals. All differences were significant with *P*<0.001 (Student's *t-*test).
(TIF)

Figure S3 (A) Comparative RACE analysis of the 3′-terminal region of *Fbn2* identified a Wt-*Fbn2* amplicon of the predicted size present in the Wt transcript (arrow) but absent in *Mp/Mp*; and a smaller amplicon that was present in *Mp/Mp* (arrowhead) but absent from Wt. (B) RT-PCR using primers specific for the 3′-ends of each *Fbn2* and *Isoc1* failed to amplify products from homozygote cDNA. However, using different combinations of these primers (e.g. *Fbn2* 3′-fwd with the *Isoc1* reverse primer; or the *Isoc1*-3′-fwd primer with a *Fbn2* reverse) confirmed the reciprocal fusion between *Fbn2* and *Isoc1* mRNAs prepared from MEF cultures. *Slc27a6* expression was unaffected. Bottom panel is control RT-PCR for *ß-Actin*. (C) *Isoc1* and *Slc27a6* expression by quantitative RT-PCR using cDNA prepared from E13.5 eyes. *Isoc1* mRNA was ∼50% reduced in *Mp/Mp* compared to Wt, whereas *Slc27a6* mRNA levels showed no difference. (D) H&E-stained eye sections showed that *Fbn2^tm1rmz/tm1rmz^* eyes from the 6th back-cross to C57BL/6J, displayed no structural abnormalities at P0, whereas stage-matched *Mp/Mp* eyes displayed clearly identifiable overall size reduction, absent vitreous, thinned non-pigmented ciliary margin (arrowheads), and some retinal-layer disruption (arrows). (E) Similarly, adult stage P21 *Fbn2^tm1rmz/tm1rmz^* retinas on a 129/Sv background displayed no structural abnormalities (compare to Wt & *Mp* P21 eye sections in Figure 2).
(TIF)

Figure S4 (A) Reciprocal in situ hybridisation of E11.5 embryos for *Fbn2*, *Isoc1* and *Slc27a6* showed ocular (arrowhead), limb (red arrowhead) and somite (arrows) expression of *Fbn2* in Wt and *Mp/Mp* embryos. Ocular expression from digital coronal sections of *Isoc1* and *Fbn2* is also presented (red arrows). No *Isoc1* or *Slc27a6* expression was detected in either genotype. Note the 3′-riboprobes were used reciprocally, with the *Fbn2* and *Isoc1* probes acting as useful positive controls. *n* = 3 embryos per genotype for each RNA probe. (B) Further reciprocal in situ hybridisation of E14.5 hind limbs for *Fbn2* and *Isoc1* showed that spatial and temporal expression of *Fbn2* and *Fbn2^Mp^* was unaffected by the *Mp* mutation. (C) Immunostaining of primary MEF cultures established from wild type and *Mp/Mp* embryos with pAb868 revealed differences in Fbn2 localisation. *Mp* Fbn2 was intracellular, whereas Wt appeared extracellular. (D) Similarly, Wt hind-limb articular cartilage revealed typical extracellular microfibrillar localisation of Fibrillin-2. Arrowheads in inset indicate the Fibrillin-2 positive microfibrils in a higher magnification image. In contrast, Fibrillin-2^Mp^ in equivalent mutant tissue was identified in discrete foci (arrows in inset) adjacent to cell nuclei (DAPI, blue stain) and appeared intracellular. (E) *Top*: Analysis of neonatal skin sections with pAb868 for Fibrillin-2 localisation revealed differential staining between genotypes, with extracellular protein organised into microfibrils in Wt dermis but not in *Mp/Mp*, where the mutant protein was observed in discrete foci and not observed extracellularly. *Bottom*: Equivalent staining for Fibrillin-1 localisation displayed comparable extracellular localisation in Wt and *Mp*, and no intracellular localisation was observed. Similar results were observed with perichondrium and nerve tissue (data not shown). Scale bars = 50 µm. Abbreviations: hf, hair follicle.
(TIF)

Figure S5 (A) Immunostaining for PDI protein (dark, colourometric stain) was not strongly observed in Wt eyes, but in the developing *Mp/Mp* eye (B) PDI was spatially consistent with Fbn2^Mp^ inclusions at the distal retina (arrows) and adjacent rpe. (C) Fbn2^Mp^ (green) and PDI colocalisation was observed at the anterior neural retina and RPE in *Mp/Mp* cells using serial immunohistochemistry. (D–G) Anti-activated caspase-3 antibody staining in Wt anterior and central retina revealed no apoptotic cells. In contrast, staining of retinal sections at anterior or central regions revealed an increase in signal foci (arrowheads) in *Mp/Mp* eyes, but that positive cells were distributed throughout the retina and were not specific to the anterior region of the developing *Mp* eye.
(TIF)

Figure S6 Conditioned media and cell lysates from Wt human RPE1 cultures transiently transfected with Wnt-FLAG and chemically induced for ER-stress with tunicamycin treatment (Tn) were collected and immunoblotting was performed. In the conditioned media from the Tn-treated cultures, both signal intensity and band migration of Wnt-FLAG were reduced compared to the untreated cultures, consistent with the inhibition to both glycosylation and secretion. In the cell lysates however, only the size of the migrated protein was different between samples. Immunoblotting of cell lysates with anti-Hspa5 antibody confirmed the induction of ER-stress in the Tn treated samples.
(TIF)

Table S1 Oligonucleotide primers used in this study.
(DOCX)

Table S2 Primary antibodies used in this study.
(DOCX)

## References

[1] Worman HJ, Fong LG, Muchir A, Young SG (2009) Laminopathies and the long strange trip from basic cell biology to therapy. J Clin Invest 119: 1825–1836. 19587457 10.1172/JCI37679 PMC2701866

[2] Robertson SP (2005) Filamin A: phenotypic diversity. Curr Opin Genet Dev 15: 301–307. 15917206 10.1016/j.gde.2005.04.001

[3] Wilkie AO (2005) Bad bones, absent smell, selfish testes: the pleiotropic consequences of human FGF receptor mutations. Cytokine Growth Factor Rev 16: 187–203. 15863034 10.1016/j.cytogfr.2005.03.001

[4] Roussel BD KA, Miranda E, Crowther DC, Lomas DA, Marciniak SJ (2013) Endoplasmic reticulum dysfunction in neurological disease. Lancet Neurol 12: 105–118. 23237905 10.1016/S1474-4422(12)70238-7

[5] Xia K, Ma H, Xiong H, Pan Q, Huang L, et al (2010) Trafficking abnormality and ER stress underlie functional deficiency of hearing impairment-associated connexin-31 mutants. Protein Cell 1: 935–943. 21204020 10.1007/s13238-010-0118-7 PMC4875122

[6] Tsang KY, Chan D, Cheslett D, Chan WC, So CL, et al (2007) Surviving endoplasmic reticulum stress is coupled to altered chondrocyte differentiation and function. PLoS Biol 5: e44. 17298185 10.1371/journal.pbio.0050044 PMC1820825

[7] Vij N, Fang S, Zeitlin PL (2006) Selective inhibition of endoplasmic reticulum-associated degradation rescues DeltaF508-cystic fibrosis transmembrane regulator and suppresses interleukin-8 levels: therapeutic implications. J Biol Chem 281: 17369–17378. 16621797 10.1074/jbc.M600509200

[8] Morrison D, Fitzpatrick D, Hanson I, Williamson K, van Heyningen V, et al (2002) National study of microphthalmia, anophthalmia, and coloboma (MAC) in Scotland: investigation of genetic aetiology. Journal of Medical Genetics 39: 16–22. 11826019 10.1136/jmg.39.1.16 PMC1734963

[9] Stoll C, Alembik Y, Dott B, Roth MP (1997) Congenital eye malformations in 212,479 consecutive births. Ann Genet 40: 122–128. 9259960

[10] Fitzpatrick DR, van Heyningen V (2005) Developmental eye disorders. Curr Opin Genet Dev 15: 348–353. 15917212 10.1016/j.gde.2005.04.013

[11] Fantes J, Ragge NK, Lynch SA, McGill NI, Collin JR, et al (2003) Mutations in SOX2 cause anophthalmia. Nat Genet 33: 461–463. 12612584 10.1038/ng1120

[12] Ragge NK, Lorenz B, Schneider A, Bushby K, de Sanctis L, et al (2005) SOX2 anophthalmia syndrome. Am J Med Genet A 135: 1–7 discussion 8. 15812812 10.1002/ajmg.a.30642

[13] Ragge NK, Brown AG, Poloschek CM, Lorenz B, Henderson RA, et al (2005) Heterozygous mutations of OTX2 cause severe ocular malformations. Am J Hum Genet 76: 1008–1022. 15846561 10.1086/430721 PMC1196439

[14] Pasutto F, Sticht H, Hammersen G, Gillessen-Kaesbach G, Fitzpatrick DR, et al (2007) Mutations in STRA6 cause a broad spectrum of malformations including anophthalmia, congenital heart defects, diaphragmatic hernia, alveolar capillary dysplasia, lung hypoplasia, and mental retardation. Am J Hum Genet 80: 550–560. 17273977 10.1086/512203 PMC1821097

[15] Reis LM, Tyler RC, Schneider A, Bardakjian T, Stoler JM, et al (2010) FOXE3 plays a significant role in autosomal recessive microphthalmia. Am J Med Genet A 152A: 582–590. 20140963 10.1002/ajmg.a.33257 PMC2998041

[16] Abouzeid H, Boisset G, Favez T, Youssef M, Marzouk I, et al (2011) Mutations in the SPARC-related modular calcium-binding protein 1 gene, SMOC1, cause waardenburg anophthalmia syndrome. Am J Hum Genet 88: 92–98. 21194680 10.1016/j.ajhg.2010.12.002 PMC3014360

[17] Okada I, Hamanoue H, Terada K, Tohma T, Megarbane A, et al (2011) SMOC1 is essential for ocular and limb development in humans and mice. Am J Hum Genet 88: 30–41. 21194678 10.1016/j.ajhg.2010.11.012 PMC3014372

[18] Rainger J, van Beusekom E, Ramsay JK, McKie L, Al-Gazali L, et al (2011) Loss of the BMP antagonist, SMOC-1, causes Ophthalmo-acromelic (Waardenburg Anophthalmia) syndrome in humans and mice. PLoS Genet 7: e1002114. 21750680 10.1371/journal.pgen.1002114 PMC3131273

[19] Glaser T, Jepeal L, Edwards JG, Young SR, Favor J, et al (1994) PAX6 gene dosage effect in a family with congenital cataracts, aniridia, anophthalmia and central nervous system defects. Nat Genet 7: 463–471. 7951315 10.1038/ng0894-463

[20] Gerth-Kahlert C WK, Ansari M, Rainger J, Hignst V, Zimmermann T, et al (2013) Clinical and Mutation Analysis of 51 Probands with Anophthalmia and/or Severe Microphthalmia from a Single Centre. Molecular Genetics & Genomic Medicine 1: 15–31. 24498598 10.1002/mgg3.2 PMC3893155

[21] Furuta Y, Hogan BL (1998) BMP4 is essential for lens induction in the mouse embryo. Genes Dev 12: 3764–3775. 9851982 10.1101/gad.12.23.3764 PMC317259

[22] Kamachi Y, Uchikawa M, Collignon J, Lovell-Badge R, Kondoh H (1998) Involvement of Sox1, 2 and 3 in the early and subsequent molecular events of lens induction. Development 125: 2521–2532. 9609835 10.1242/dev.125.13.2521

[23] Matsushima D, Heavner W, Pevny LH (2011) Combinatorial regulation of optic cup progenitor cell fate by SOX2 and PAX6. Development 138: 443–454. 21205789 10.1242/dev.055178 PMC3014633

[24] Beebe DC (1986) Development of the ciliary body: a brief review. Trans Ophthalmol Soc U K 105 (Pt 2) 123–130. 3541302

[25] Agathocleous M, Iordanova I, Willardsen MI, Xue XY, Vetter ML, et al (2009) A directional Wnt/beta-catenin-Sox2-proneural pathway regulates the transition from proliferation to differentiation in the Xenopus retina. Development 136: 3289–3299. 19736324 10.1242/dev.040451 PMC2739145

[26] Van Raay TJ, Moore KB, Iordanova I, Steele M, Jamrich M, et al (2005) Frizzled 5 signaling governs the neural potential of progenitors in the developing Xenopus retina. Neuron 46: 23–36. 15820691 10.1016/j.neuron.2005.02.023

[27] Cho SH, Cepko CL (2006) Wnt2b/beta-catenin-mediated canonical Wnt signaling determines the peripheral fates of the chick eye. Development 133: 3167–3177. 16854977 10.1242/dev.02474

[28] Taranova OV, Magness ST, Fagan BM, Wu Y, Surzenko N, et al (2006) SOX2 is a dose-dependent regulator of retinal neural progenitor competence. Genes Dev 20: 1187–1202. 16651659 10.1101/gad.1407906 PMC1472477

[29] Phipps E (1964) Mp. Mouse News Letter 31: 41.

[30] Arteaga-Solis E, Gayraud B, Lee SY, Shum L, Sakai L, et al (2001) Regulation of limb patterning by extracellular microfibrils. J Cell Biol 154: 275–281. 11470817 10.1083/jcb.200105046 PMC2150751

[31] Chaudhry SS, Gazzard J, Baldock C, Dixon J, Rock MJ, et al (2001) Mutation of the gene encoding fibrillin-2 results in syndactyly in mice. Hum Mol Genet 10: 835–843. 11285249 10.1093/hmg/10.8.835

[32] Miller G, Neilan M, Chia R, Gheryani N, Holt N, et al (2010) ENU mutagenesis reveals a novel phenotype of reduced limb strength in mice lacking fibrillin 2. PLoS One 5: e9137. 20161761 10.1371/journal.pone.0009137 PMC2817753

[33] Maretto S, Cordenonsi M, Dupont S, Braghetta P, Broccoli V, et al (2003) Mapping Wnt/beta-catenin signaling during mouse development and in colorectal tumors. Proc Natl Acad Sci U S A 100: 3299–3304. 12626757 10.1073/pnas.0434590100 PMC152286

[34] Johnson KR, Cook SA, Zheng QY (1998) The original shaker-with-syndactylism mutation (sy) is a contiguous gene deletion syndrome. Mamm Genome 9: 889–892. 9799839 10.1007/s003359900889 PMC2858217

[35] Lee B, Godfrey M, Vitale E, Hori H, Mattei MG, et al (1991) Linkage of Marfan syndrome and a phenotypically related disorder to two different fibrillin genes. Nature 352: 330–334. 1852206 10.1038/352330a0

[36] Putnam EA, Zhang H, Ramirez F, Milewicz DM (1995) Fibrillin-2 (FBN2) mutations result in the Marfan-like disorder, congenital contractural arachnodactyly. Nat Genet 11: 456–458. 7493032 10.1038/ng1295-456

[37] Wang M, Clericuzio CL, Godfrey M (1996) Familial occurrence of typical and severe lethal congenital contractural arachnodactyly caused by missplicing of exon 34 of fibrillin-2. Am J Hum Genet 59: 1027–1034. 8900230 PMC1914850

[38] Piha-Gossack A, Sossin W, Reinhardt DP (2012) The evolution of extracellular fibrillins and their functional domains. PLoS One 7: e33560. 22438950 10.1371/journal.pone.0033560 PMC3306419

[39] Chang YF, Imam JS, Wilkinson MF (2007) The nonsense-mediated decay RNA surveillance pathway. Annu Rev Biochem 76: 51–74. 17352659 10.1146/annurev.biochem.76.050106.093909

[40] Nagy E, Maquat LE (1998) A rule for termination-codon position within intron-containing genes: when nonsense affects RNA abundance. Trends Biochem Sci 23: 198–199. 9644970 10.1016/s0968-0004(98)01208-0

[41] Dixon MJ, Gazzard J, Chaudhry SS, Sampson N, Schulte BA, et al (1999) Mutation of the Na-K-Cl co-transporter gene Slc12a2 results in deafness in mice. Hum Mol Genet 8: 1579–1584. 10401008 10.1093/hmg/8.8.1579

[42] Shi Y, Tu Y, De Maria A, Mecham RP, Bassnett S (2013) Development, composition, and structural arrangements of the ciliary zonule of the mouse. Invest Ophthalmol Vis Sci 54: 2504–2515. 23493297 10.1167/iovs.13-11619 PMC3621578

[43] Keene DR, Jordan CD, Reinhardt DP, Ridgway CC, Ono RN, et al (1997) Fibrillin-1 in human cartilage: developmental expression and formation of special banded fibers. J Histochem Cytochem 45: 1069–1082. 9267468 10.1177/002215549704500805

[44] Yoshida H, Matsui T, Yamamoto A, Okada T, Mori K (2001) XBP1 mRNA is induced by ATF6 and spliced by IRE1 in response to ER stress to produce a highly active transcription factor. Cell 107: 881–891. 11779464 10.1016/s0092-8674(01)00611-0

[45] Zhang H, Hu W, Ramirez F (1995) Developmental expression of fibrillin genes suggests heterogeneity of extracellular microfibrils. J Cell Biol 129: 1165–1176. 7744963 10.1083/jcb.129.4.1165 PMC2120487

[46] Quondamatteo F, Reinhardt DP, Charbonneau NL, Pophal G, Sakai LY, et al (2002) Fibrillin-1 and fibrillin-2 in human embryonic and early fetal development. Matrix Biol 21: 637–646. 12524050 10.1016/s0945-053x(02)00100-2

[47] Hilhorst-Hofstee Y, Hamel BC, Verheij JB, Rijlaarsdam ME, Mancini GM, et al (2011) The clinical spectrum of complete FBN1 allele deletions. Eur J Hum Genet 19: 247–252. 21063442 10.1038/ejhg.2010.174 PMC3061999

[48] Faivre L, Collod-Beroud G, Callewaert B, Child A, Binquet C, et al (2009) Clinical and mutation-type analysis from an international series of 198 probands with a pathogenic FBN1 exons 24–32 mutation. Eur J Hum Genet 17: 491–501. 19002209 10.1038/ejhg.2008.207 PMC2734964

[49] Park ES, Putnam EA, Chitayat D, Child A, Milewicz DM (1998) Clustering of FBN2 mutations in patients with congenital contractural arachnodactyly indicates an important role of the domains encoded by exons 24 through 34 during human development. Am J Med Genet 78: 350–355. 9714438

[50] Goldblatt J, Hyatt J, Edwards C, Walpole I (2011) Further evidence for a marfanoid syndrome with neonatal progeroid features and severe generalized lipodystrophy due to frameshift mutations near the 3′ end of the FBN1 gene. Am J Med Genet A 155A: 717–720. 21594992 10.1002/ajmg.a.33906

[51] Graul-Neumann LM, Kienitz T, Robinson PN, Baasanjav S, Karow B, et al (2010) Marfan syndrome with neonatal progeroid syndrome-like lipodystrophy associated with a novel frameshift mutation at the 3′ terminus of the FBN1-gene. Am J Med Genet A 152A: 2749–2755. 20979188 10.1002/ajmg.a.33690

[52] Horn D, Robinson PN (2011) Progeroid facial features and lipodystrophy associated with a novel splice site mutation in the final intron of the FBN1 gene. Am J Med Genet A 155A: 721–724. 21594993 10.1002/ajmg.a.33905

[53] Ritty TM, Broekelmann T, Tisdale C, Milewicz DM, Mecham RP (1999) Processing of the fibrillin-1 carboxyl-terminal domain. J Biol Chem 274: 8933–8940. 10085138 10.1074/jbc.274.13.8933

[54] Tsang KY, Chan D, Bateman JF, Cheah KS (2010) In vivo cellular adaptation to ER stress: survival strategies with double-edged consequences. J Cell Sci 123: 2145–2154. 20554893 10.1242/jcs.068833

[55] Bishop PN, Takanosu M, Le Goff M, Mayne R (2002) The role of the posterior ciliary body in the biosynthesis of vitreous humour. Eye (Lond) 16: 454–460. 12101453 10.1038/sj.eye.6700199

[56] Halfter W, Dong S, Schurer B, Ring C, Cole GJ, et al (2005) Embryonic synthesis of the inner limiting membrane and vitreous body. Invest Ophthalmol Vis Sci 46: 2202–2209. 15914642 10.1167/iovs.04-1419

[57] Liu H, Mohamed O, Dufort D, Wallace VA (2003) Characterization of Wnt signaling components and activation of the Wnt canonical pathway in the murine retina. Dev Dyn 227: 323–334. 12815618 10.1002/dvdy.10315

[58] Nakagawa S, Takada S, Takada R, Takeichi M (2003) Identification of the laminar-inducing factor: Wnt-signal from the anterior rim induces correct laminar formation of the neural retina in vitro. Dev Biol 260: 414–425. 12921742 10.1016/s0012-1606(03)00320-8

[59] Maddox BK, Keene DR, Sakai LY, Charbonneau NL, Morris NP, et al (1997) The fate of cartilage oligomeric matrix protein is determined by the cell type in the case of a novel mutation in pseudoachondroplasia. J Biol Chem 272: 30993–30997. 9388247 10.1074/jbc.272.49.30993

[60] Merritt TM, Bick R, Poindexter BJ, Alcorn JL, Hecht JT (2007) Unique matrix structure in the rough endoplasmic reticulum cisternae of pseudoachondroplasia chondrocytes. Am J Pathol 170: 293–300. 17200202 10.2353/ajpath.2007.060530 PMC1762700

[61] Dinser R, Zaucke F, Kreppel F, Hultenby K, Kochanek S, et al (2002) Pseudoachondroplasia is caused through both intra- and extracellular pathogenic pathways. J Clin Invest 110: 505–513. 12189245 10.1172/JCI14386 PMC150414

[62] Suleman F, Gualeni B, Gregson HJ, Leighton MP, Pirog KA, et al (2012) A novel form of chondrocyte stress is triggered by a COMP mutation causing pseudoachondroplasia. Hum Mutat 33: 218–231. 22006726 10.1002/humu.21631 PMC3320758

[63] Harewood L, Liu M, Keeling J, Howatson A, Whiteford M, et al (2010) Bilateral renal agenesis/hypoplasia/dysplasia (BRAHD): postmortem analysis of 45 cases with breakpoint mapping of two de novo translocations. PLoS One 5: e12375. 20811621 10.1371/journal.pone.0012375 PMC2928268

[64] Wilson R, Diseberg AF, Gordon L, Zivkovic S, Tatarczuch L, et al (2010) Comprehensive profiling of cartilage extracellular matrix formation and maturation using sequential extraction and label-free quantitative proteomics. Mol Cell Proteomics 9: 1296–1313. 20190199 10.1074/mcp.M000014-MCP201 PMC2877988

[65] Asara JM, Christofk HR, Freimark LM, Cantley LC (2008) A label-free quantification method by MS/MS TIC compared to SILAC and spectral counting in a proteomics screen. Proteomics 8: 994–999. 18324724 10.1002/pmic.200700426

