# Supplementary figures and images for: A Trans-Acting Protein Effect Causes Severe Eye Malformation in the Mp Mouse
Source: PLoS Genet. 2013 Dec 12;9(12):e1003998. doi: 10.1371/journal.pgen.1003998 (PMC3861116; doi:10.1371/journal.pgen.1003998)

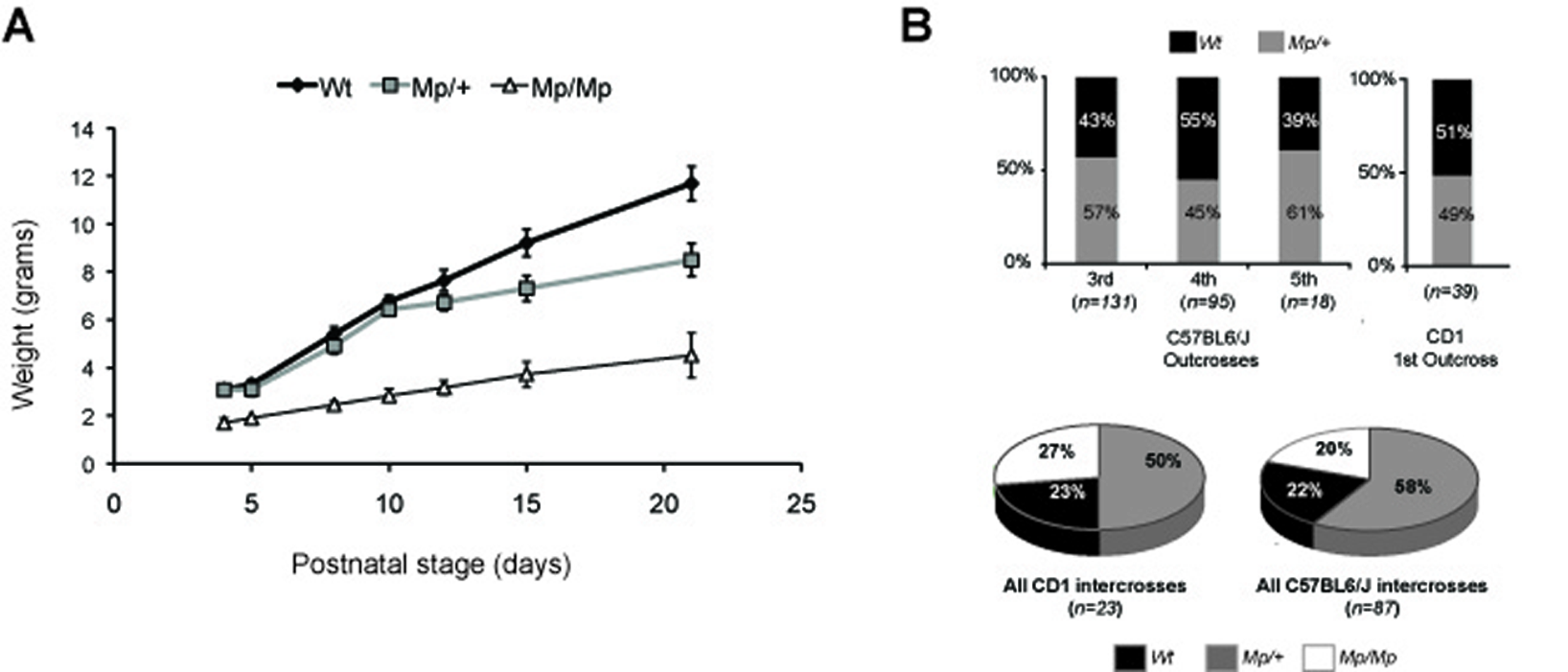

Supplement: Figure S1 — (A) Average weights (in grams) recorded between postnatal days 4 and P21 showed reduced weight of Mp/Mp from P4 compared to Wt and Mp/+. In addition, both mutant types failed to gain weight compared to Wt. Error bars, s.d. (B) Phenotype data from out-crosses (top) and intercrosses (bottom) to C57BL6/J and CD1 genetic backgrounds revealed that the Mp phenotype was fully penetrant on both strains. (TIF) [file pgen.1003998.s001.tif]

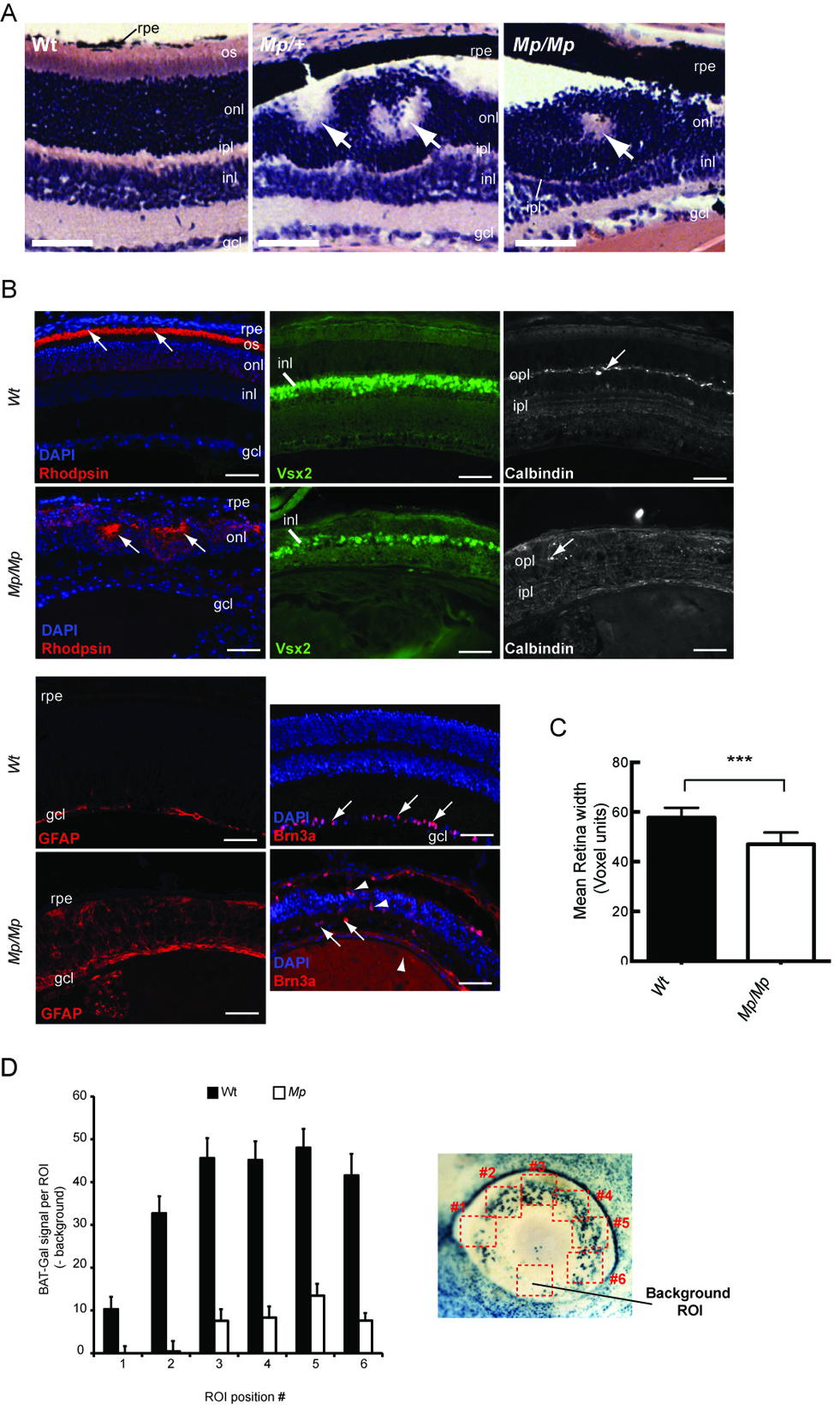

Supplement: Figure S2 — (A) Haemotoxylin and eosin staining of P21 retinal tissue revealed retinal rosetting affecting the inner nuclear layer cells (arrows). Scale bar, 100 µm. (B) Immunohistochemical marker analysis revealed gross disruption to Mp retinas at P21. Staining for Rhodopsin was localised to punctate regions of the mutant retina (arrows), consistent with rosette foci, and was not identified in the outer segment of the retina, in contrast to the Wt retina where Rhodopsin signal was localised to the outer segment (arrows) and the outer nuclear layer (arrowheads). DAPI counterstaining illustrated the disruption to the normal lamination of the Mp retina compared to Wt. Vsx2 antibody staining displayed a reduced number of cells in the Mp inner nuclear layer, and staining for Calbindin was reduced in the Mp outer plexiform layer (arrows), indicating a disruption to the projections emanating from horizontal cells of this region of the retina. GFAP (glial fibrillary acidic protein) staining, a marker for Muller glial cells and retinal astrocytes but also retinal stress responses, was markedly increased in mutant eyes. Brn3a, expressed in retinal ganglion cells (arrows) displayed ectopic staining in multiple regions of the Mp/Mp retina (arrowheads) and revealed a reduction to the typical ganglion layer compared to Wt. Abbreviations: gcl, ganglion cell layer; inl, inner nuclear layer; ipl, inner plexiform layer; is, inner segment; onl, outer nuclear layer; opl, outer plexiform layer; os, outer segment; rpe, retinal pigmented epithelium. Scale bar = 100 µm. (C) Mean retinal widths at E15.5 measured in voxel units using OPT data from whole embryonic heads. Error bars, s.d. Wt n = 5; Mp/Mp n = 8. ***P<0.001 (Student's t-test). (D) Quantitative ß-galactosidase staining of Wt:BAT-Gal and MpMp:BAT-Gal eyes at E14.5 was performed by the segmentation of the eye into 6 regions of interest (ROI) and staining intensity was measured for each ROI using Photoshop software (Adobe) and normalised to th [file pgen.1003998.s002.tif]

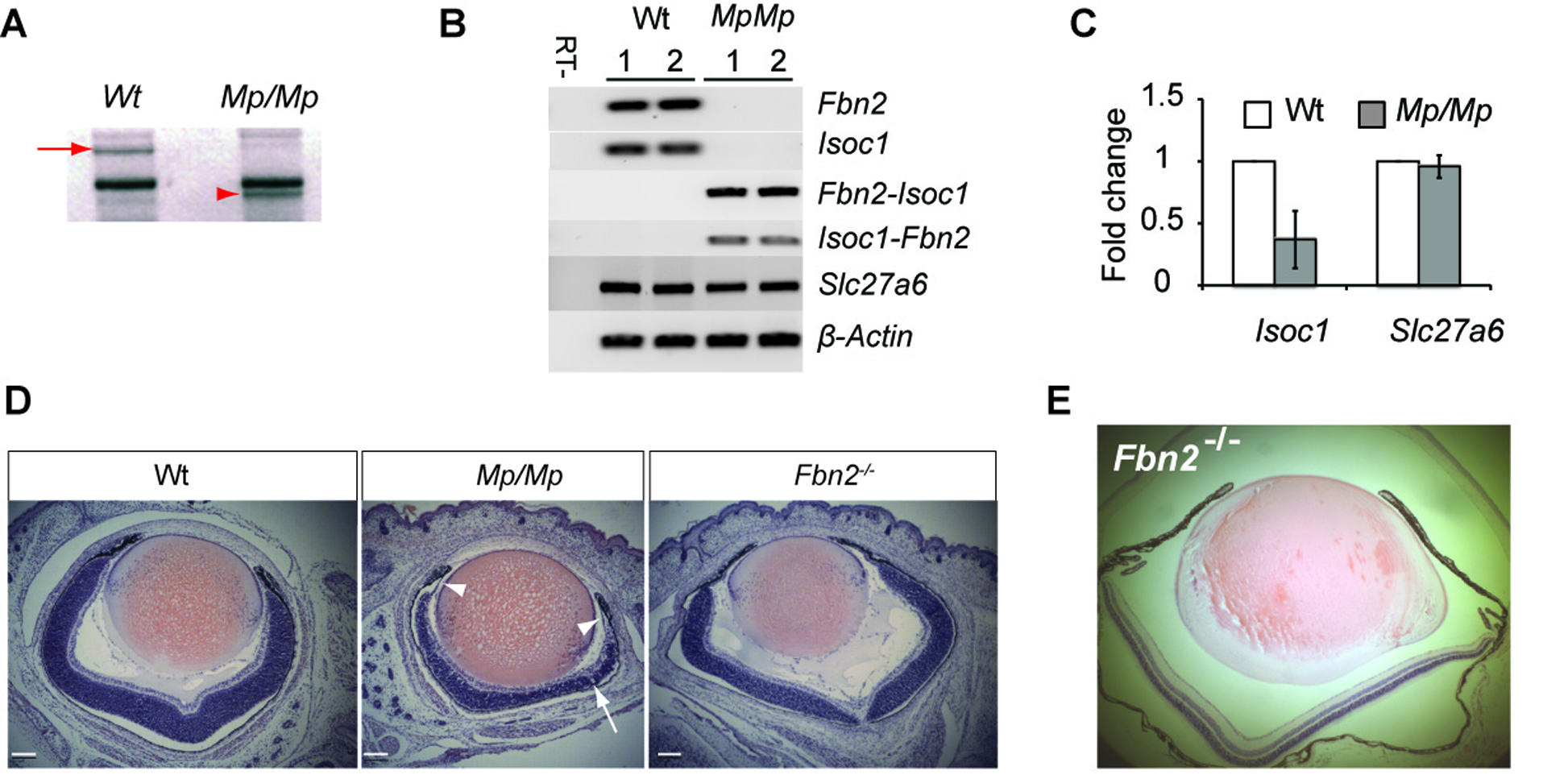

Supplement: Figure S3 — (A) Comparative RACE analysis of the 3′-terminal region of Fbn2 identified a Wt-Fbn2 amplicon of the predicted size present in the Wt transcript (arrow) but absent in Mp/Mp; and a smaller amplicon that was present in Mp/Mp (arrowhead) but absent from Wt. (B) RT-PCR using primers specific for the 3′-ends of each Fbn2 and Isoc1 failed to amplify products from homozygote cDNA. However, using different combinations of these primers (e.g. Fbn2 3′-fwd with the Isoc1 reverse primer; or the Isoc1-3′-fwd primer with a Fbn2 reverse) confirmed the reciprocal fusion between Fbn2 and Isoc1 mRNAs prepared from MEF cultures. Slc27a6 expression was unaffected. Bottom panel is control RT-PCR for ß-Actin. (C) Isoc1 and Slc27a6 expression by quantitative RT-PCR using cDNA prepared from E13.5 eyes. Isoc1 mRNA was ∼50% reduced in Mp/Mp compared to Wt, whereas Slc27a6 mRNA levels showed no difference. (D) H&E-stained eye sections showed that Fbn2tm1rmz/tm1rmz eyes from the 6th back-cross to C57BL/6J, displayed no structural abnormalities at P0, whereas stage-matched Mp/Mp eyes displayed clearly identifiable overall size reduction, absent vitreous, thinned non-pigmented ciliary margin (arrowheads), and some retinal-layer disruption (arrows). (E) Similarly, adult stage P21 Fbn2tm1rmz/tm1rmz retinas on a 129/Sv background displayed no structural abnormalities (compare to Wt & Mp P21 eye sections in Figure 2). (TIF) [file pgen.1003998.s003.tif]

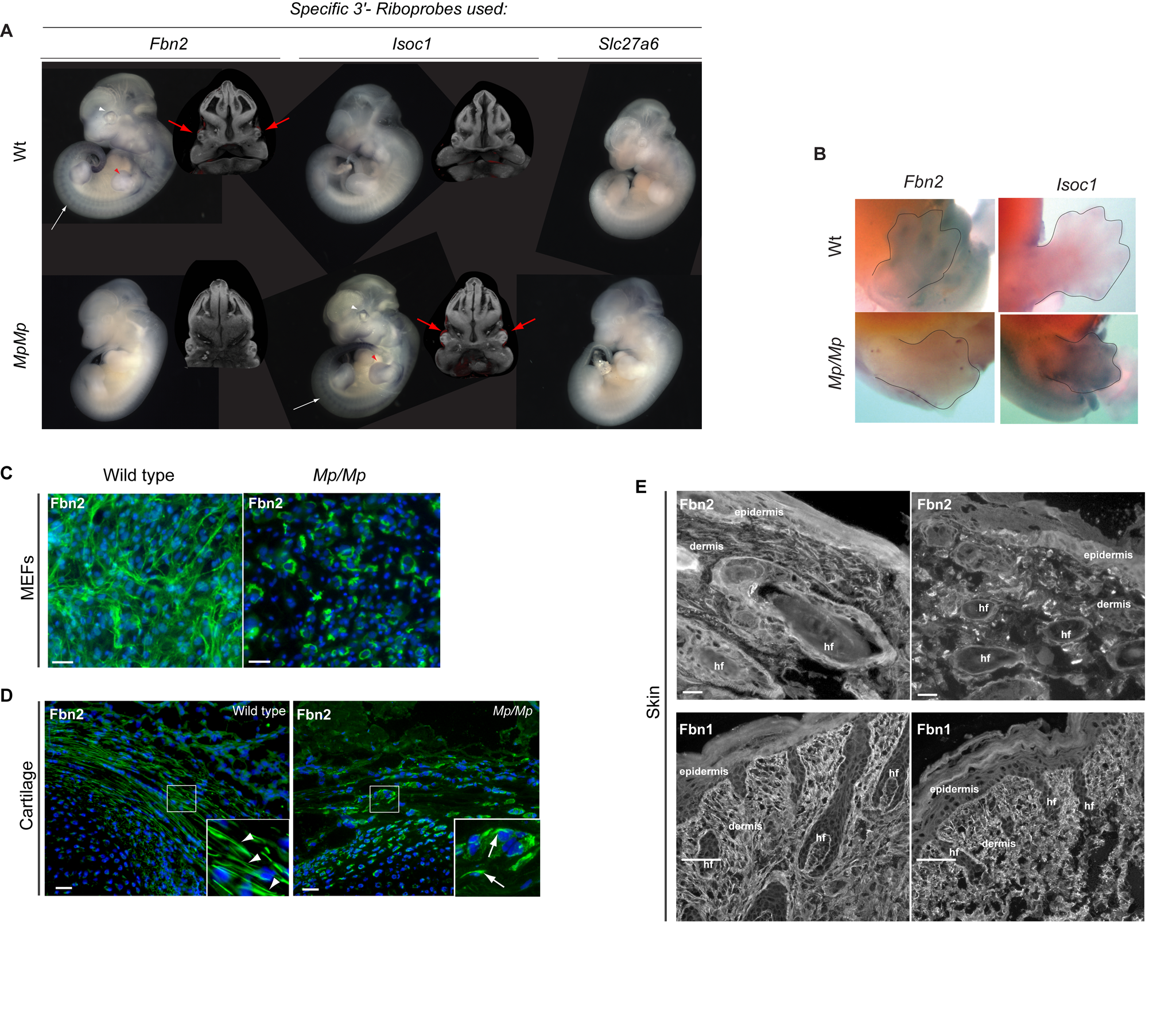

Supplement: Figure S4 — (A) Reciprocal in situ hybridisation of E11.5 embryos for Fbn2, Isoc1 and Slc27a6 showed ocular (arrowhead), limb (red arrowhead) and somite (arrows) expression of Fbn2 in Wt and Mp/Mp embryos. Ocular expression from digital coronal sections of Isoc1 and Fbn2 is also presented (red arrows). No Isoc1 or Slc27a6 expression was detected in either genotype. Note the 3′-riboprobes were used reciprocally, with the Fbn2 and Isoc1 probes acting as useful positive controls. n = 3 embryos per genotype for each RNA probe. (B) Further reciprocal in situ hybridisation of E14.5 hind limbs for Fbn2 and Isoc1 showed that spatial and temporal expression of Fbn2 and Fbn2Mp was unaffected by the Mp mutation. (C) Immunostaining of primary MEF cultures established from wild type and Mp/Mp embryos with pAb868 revealed differences in Fbn2 localisation. Mp Fbn2 was intracellular, whereas Wt appeared extracellular. (D) Similarly, Wt hind-limb articular cartilage revealed typical extracellular microfibrillar localisation of Fibrillin-2. Arrowheads in inset indicate the Fibrillin-2 positive microfibrils in a higher magnification image. In contrast, Fibrillin-2Mp in equivalent mutant tissue was identified in discrete foci (arrows in inset) adjacent to cell nuclei (DAPI, blue stain) and appeared intracellular. (E) Top: Analysis of neonatal skin sections with pAb868 for Fibrillin-2 localisation revealed differential staining between genotypes, with extracellular protein organised into microfibrils in Wt dermis but not in Mp/Mp, where the mutant protein was observed in discrete foci and not observed extracellularly. Bottom: Equivalent staining for Fibrillin-1 localisation displayed comparable extracellular localisation in Wt and Mp, and no intracellular localisation was observed. Similar results were observed with perichondrium and nerve tissue (data not shown). Scale bars = 50 µm. Abbreviations: hf, hair follicle. (TIF) [file pgen.1003998.s004.tif]

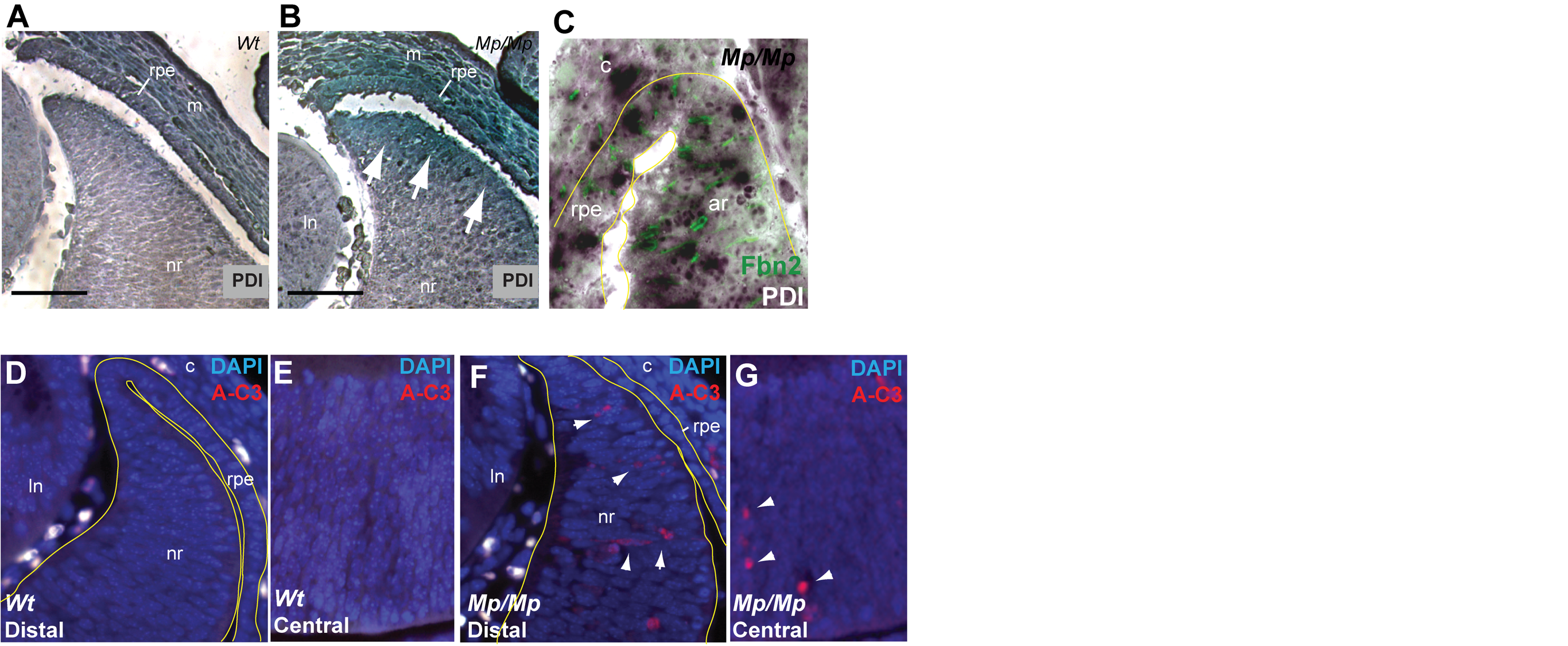

Supplement: Figure S5 — (A) Immunostaining for PDI protein (dark, colourometric stain) was not strongly observed in Wt eyes, but in the developing Mp/Mp eye (B) PDI was spatially consistent with Fbn2Mp inclusions at the distal retina (arrows) and adjacent rpe. (C) Fbn2Mp (green) and PDI colocalisation was observed at the anterior neural retina and RPE in Mp/Mp cells using serial immunohistochemistry. (D–G) Anti-activated caspase-3 antibody staining in Wt anterior and central retina revealed no apoptotic cells. In contrast, staining of retinal sections at anterior or central regions revealed an increase in signal foci (arrowheads) in Mp/Mp eyes, but that positive cells were distributed throughout the retina and were not specific to the anterior region of the developing Mp eye. (TIF) [file pgen.1003998.s005.tif]

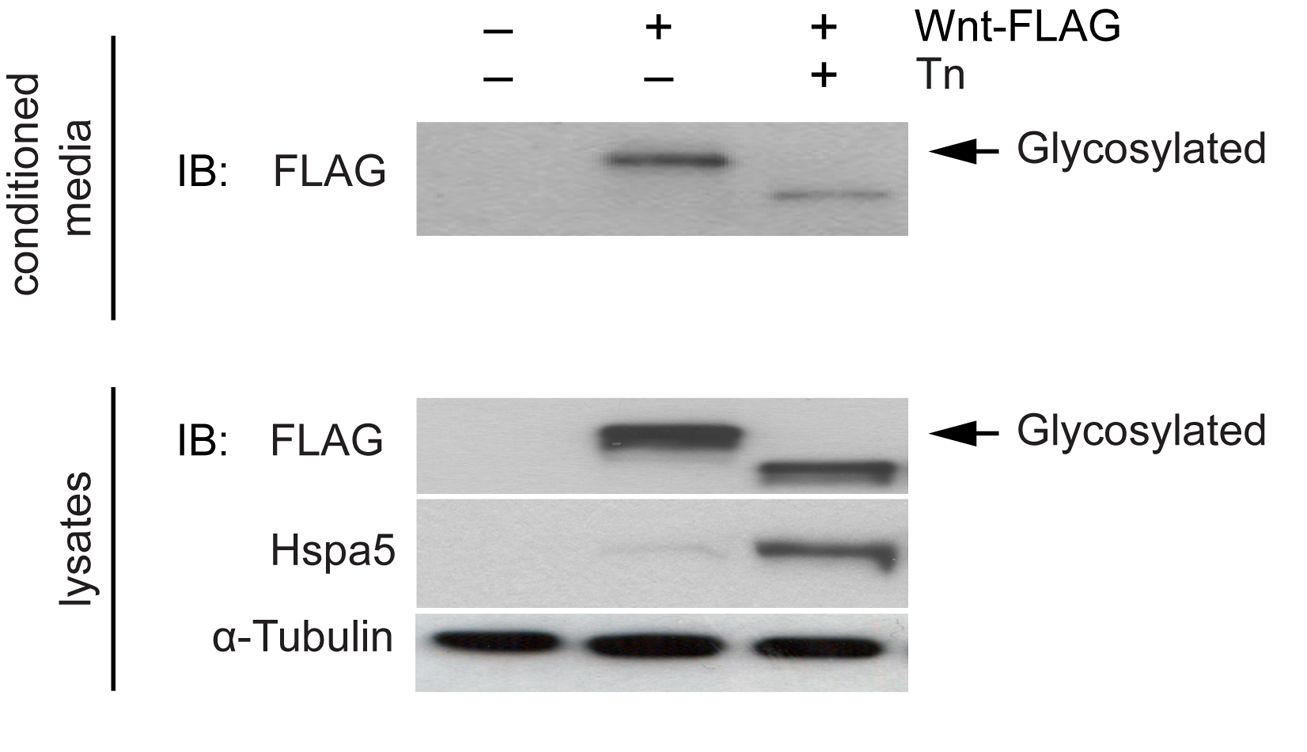

Supplement: Figure S6 — Conditioned media and cell lysates from Wt human RPE1 cultures transiently transfected with Wnt-FLAG and chemically induced for ER-stress with tunicamycin treatment (Tn) were collected and immunoblotting was performed. In the conditioned media from the Tn-treated cultures, both signal intensity and band migration of Wnt-FLAG were reduced compared to the untreated cultures, consistent with the inhibition to both glycosylation and secretion. In the cell lysates however, only the size of the migrated protein was different between samples. Immunoblotting of cell lysates with anti-Hspa5 antibody confirmed the induction of ER-stress in the Tn treated samples. (TIF) [file pgen.1003998.s006.tif]
