# Supplementary material for: A Trans-Acting Protein Effect Causes Severe Eye Malformation in the Mp Mouse
Source: PLoS Genet. 2013 Dec 12;9(12):e1003998. doi: 10.1371/journal.pgen.1003998 (PMC3861116; doi:10.1371/journal.pgen.1003998)
Supplement: Table S1 — Oligonucleotide primers used in this study. (DOCX) [file pgen.1003998.s007.docx]

***Supplemental Table S1***

|  | **Primer name** | **5'-3' prime sequence** | **Modificatiion** |  |
| --- | --- | --- | --- | --- |
| **Chromosome 18 mapping** | D18MIT94_Fwd | TCACCTAGGACCCCCCTC |  |  |
|  | D18MIT94_Rev | AAGTAGTGAGAGGCCACCACA |  |  |
|  | D18MIT64_Fwd | TCAGATTCACTGCTAAGTCTTTTC | 5'-6-FAM |  |
|  | D18MIT64_Rev | AGCAAGAAAAGCAGGTGAGG |  |  |
|  | D18MIT116_Fwd | CCTTAAAGGAGTGTGTATATTTTTGTG | 5'-HEX |  |
|  | D18MIT116_Rev | TTGATGTTATCCTCTGGGCC |  |  |
|  | D18MIT226_Fwd | 8CAGGCAGGGTGCATATATTATAA | 5'-HEX |  |
|  | D18MIT226_Rev | TATCTGTTTATGTGTGTACATTGTGTG |  |  |
|  | D18MIT74_Fwd | AGCCAGAGCTACAAAGTTTCAA |  |  |
|  | D18MIT74_Rev | GCTCTTGTAGAGCCATCATTCC |  |  |
|  | D18MIT184_Fwd | CACACATGTGTAGGTAGGTAGGTAGG | 5'-TET |  |
|  | D18MIT184_Rev | CGCACAAGGACTACTGAAACA |  |  |
| Fbn2 transcript analysis | FBN2cDNA1_Fwd | TCTCCAGCCCTACTTCGTGT |  |  |
|  | FBN2cDNA1_Rev | GCTGGACACATCTCACAAGG |  |  |
|  | FBN2cDNA2_Fwd | GCCCGTGTTTCACTCAAGTC |  |  |
|  | FBN2cDNA2_Rev | CCATTACTGCAGGGATTGGA |  |  |
|  | FBN2cDNA3_Fwd | GATCCTTCCGCTGTGACTGT |  |  |
|  | FBN2cDNA3_Fwd | AGGAAGCAGTGCTCCATACG |  |  |
|  | FBN2cDNA3_Rev | AGGAAGCAGTGCTCCATACG |  |  |
|  | FBN2cDNA4_Fwd | AATGAATGCGAGGTGTTTCC |  |  |
|  | FBN2cDNA4_Rev | CGTAGCTTCCTTCGGAGTTG |  |  |
|  | FBN2cDNA5_Fwd | TGATCGGAACTTACCAGTGCT |  |  |
|  | FBN2cDNA5_Rev | GTTTCCTCCTGTCCTGTCCA |  |  |
|  | FBN2cDNA6_Fwd | AGTGTGAGATGGGCTTCACC |  |  |
|  | FBN2cDNA6_Rev | TGTCAAAGGTGAATCCAGGAA |  |  |
|  | FBN2cDNA7_Fwd | ACAAAGAGGATGTGCTGCTG |  |  |
|  | FBN2cDNA7_Rev | GAGCCCTCCAAATTCTGACA |  |  |
|  | FBN2cDNA8_Fwd | GCAACGAGGGTTACGAACTG |  |  |
|  | FBN2cDNA8_Rev | CCAGGCTTAGTCCTGCATTC |  |  |
|  | FBN2cDNA9_Fwd | CTGGACGAGTGTGCTGAGG |  |  |
|  | FBN2cDNA9_Rev | TCTTCACAGTTCAGCCCAGA |  |  |
|  | FBN2cDNA10_Fwd | GTCTCAGCCTTCCCTCTGTG |  |  |
|  | FBN2cDNA10_Rev | GTCGTCCTCATTGTGCTCCT |  |  |
| Fbn2 3´-exon analysis | Fbn2_Ex62_Fwd | TTTGTTCCCATTAAAGGTTGTG |  |  |
|  | Fbn2_Ex62_Rev | GCTTATCCAGACACTGAGGTTG |  |  |
|  | Fbn2_Ex63_Fwd | TTCATCCCCTCTGCCCC |  |  |
|  | Fbn2_Ex63_Rev | GCTGCCTGACCCTGACC |  |  |
|  | Fbn2_Ex64_Fwd | TGCGTCCATGTTTCCTAAAG |  |  |
|  | Fbn2_Ex64_Fwd | CAGGGCCAAGGAACACG |  |  |
|  | Fbn2_Ex64_Rev | CAGGGCCAAGGAACACG |  |  |
|  | Fbn2_Ex65_Fwd | TATCAGTCAATCGCAGGTGG |  |  |
|  | Fbn2_Ex65_Rev | CTTCTGAAGGCCGGCTG |  |  |
| Isoc1 transcript analysis | Isoc1_cDNA_Rev | TGCACAATTTAAGAATACTTCACCA |  |  |
|  | Isoc1_cDNA_Fwd | CATTAGTGTGGGACAGAGACTGTTGC |  |  |
| Mouse routine genotyping | Fbn2_FineMap_F | AGAAGCACCTTGAGCAGAGC |  |  |
|  | Fbn2_FineMap_R | GCCTTGTTTGGCTATGTTGG |  |  |
|  | Isoc1Ex5_4_Fwd2 | AGGTGATGGTGTGCAGTGTC |  |  |
| RT-PCR analysis | 18sRNA_Fwd | AGATCAAAACCAACCCGGTGA |  |  |
|  | 18sRNA_Rev | GGTAAGAGCATCGAGGGGGC |  |  |
|  | HPRT_QRTPCR_Fwd | CTGGTGAAAAGGACCTCTCG |  |  |
|  | HPRT_QRTPCR_Rev | CAAGGGCATATCCAACAACA |  |  |
|  | SLC27a6_cDNA_Fwd | TGGAATTGGTGGATGTGTTG |  |  |
|  | SLC27a6_cDNA_Rev | CGAAGTTCTCCTGGTCTTGG |  |  |
|  | SLC27a6_QRTPCR_Fwd | CTCTCCTTGGAATTGGTGGA |  |  |
|  | SLC27a6_QRTPCR_Rev | TGGTCCTTTTCCCCTTCTCT |  |  |
|  | Xbp1_SYBR_Fwd | CTGAGTCCGCAGCAGGTG |  |  |
|  | Xbp1_SYBR_Rev1 | CCAGAATGCCCAAAAGGATA |  |  |
|  | Xbp-1_Fwd_FAM | TTACGGGAGAAAACTCACGGCC | 5'-6-FAM |  |
|  | Xbp1_Rev | GGGTCCAACTTGTCCAGAAT |  |  |
| Riboprobe synthesis for whole mount in situ analyses | Isoc1_Fwd_T3 | AATTAACCCTCACTAAAGGAACAGATGGGAGTGGTTTGC |  |  |
|  | Isoc1_Rev_T7 | TAATACGACTCACTATAGGAGAGGATTCCCCTCCTTTGA |  |  |
|  | Fbn2_Fwd_T3 | AATTAACCCTCACTAAAGGCAAAAAGGGAAACTCGCTTG |  |  |
|  | Fbn2_Rev_T7 | TAATACGACTCACTATAGGAGGTGTTCCTGCTCAAAGGA |  |  |
|  | SLC27a6_Fwd_T3 | AATTAACCCTCACTAAAGGGCCTGCCCACTGTTTTTAAG |  |  |
|  | SLC27a6_Rev_T7 | TAATACGACTCACTATAGGTGGCTTTTACTTCCCAAACG |  |  |
|  | Bip_Ribo_Rev_T7 | TAATACGACTCACTATAGGTTTCTTCTGGGGCAAATGTC |  |  |
|  | Bip_Ribo_Fwd_T3 | AATTAACCCTCACTAAAGGGCTTCGTGTCTCCTCCTGAC |  |  |
| For qRT-PCR of secretion assay | Col6a1_Fwd | CGATGCAGAAGAGGTCATCA |  |  |
|  | Col6a1_Rev | CATACCCTGGTACCCGACTG |  |  |
|  | Col3a1_Fwd | AATGGCTCACCAGGACAAAG |  |  |
|  | Col3a1_Rev | CACCTGAAGGACCTCGTGTT |  |  |
|  | Col1a1_Fwd | AATGGTGCTCCTGGTATTGC |  |  |
|  | Col1a1_Rev | GGCTCCTCGTTTTCCTTCTT |  |  |
|  | Col1a2_Fwd | GTGTTCAAGGTGGCAAAGGT |  |  |
|  | Col1a2_Rev | GGCTTCCAATAGGACCAGAA |  |  |
|  | Col12a1_Fwd | ATAACTTGGGCACCTGTTGG |  |  |
|  | Col12a1_Rev | GGACCACTTTTTGGAGCTTG |  |  |
|  | PdiA3_Fwd | AGAAGCGGGTGCTTATGATG |  |  |
|  | PdiA3_Rev | TGCTGGCTGCTTTTAGGAAT |  |  |
|  | Prelamin-A/C_Fwd | CGATGAAGAGGGAAAGTTCG |  |  |
|  | Prelamin-A/C_Rev | GCCTTCCACACCAAGTCAGT |  |  |
|  | FstL1_Fwd | CCTGTGTGTGGCAGTAATGG |  |  |
|  | FstL1_Rev | CCAGCCATCTGGAATGATCT |  |  |
|  | Postn4_Fwd | ACGTCGTGGACAAACTCCTC |  |  |
|  | Postn4_Rev | GGATCTTCGTCATTGCAGGT |  |  |
|  |  |  |  |  |
|  |  |  |  |  |
|  |  |  |  |  |
|  |  |  | |  |
|  |  |  | |  |

MIT markers used for mapping

|  | |  | |  | | |  |
| --- | --- | --- | --- | --- | --- | --- | --- |
|  | |  | |  | | |  |
| **Marker** | | **C57** | | **C3H** |  |  |  |
| **D1Mit373** | | 124 | | 138 |  |  |  |
| **D1Mit403** | | 125 | | 147 |  |  |  |
| **D1MIT155** | | 247 | | 252/216 |  |  |  |
| **D2Mit365** | | 102 | | 106 |  |  |  |
| **D3Mit164** | | 135 | | 119 |  |  |  |
| **D3Mit49** | | 128 | | 110 |  |  |  |
| **D3Mit86** | | 154 | | 162 |  |  |  |
| **D4Mit124** | | 157 | | 139 |  |  |  |
| **D5MIT346.1** | | 125 | | 120 |  |  |  |
| **D5Mit135** | | 241 | | 217 |  |  |  |
| **D6Mit316** | | 95 | | 91 |  |  |  |
| **D7MIT80** | | 146 | | 148/154 |  |  |  |
| **D7Mit203** | | 146 | | 110 |  |  |  |
| **D8Mit200** | | 197 | | 217 |  |  |  |
| **D10Mit206** | | 148 | | 134 |  |  |  |
| **D10MIT189** | | 105 | | 130 |  |  |  |
| **D10Mit223** | | 150 | | - |  |  |  |
| **D11MIT226** | | 140 | | 142/126 |  |  |  |
| **D12Mit153** | | 142 | | 156 |  |  |  |
| **D12Mit14** | | 130 | | 146 |  |  |  |
| **D12MIT29** | | 259 | | 264 |  |  |  |
| **D13MIT17** | | 170 | | 172/154 |  |  |  |
| **D13Mit253** | | 78 | | 100 |  |  |  |
| **D13Mit76** | | 106 | | 88 |  |  |  |
| **D14Mit62** | | 120 | | 134 |  |  |  |
| **D15MIT265** | | 114 | | 118 |  |  |  |
| **D16Mit189** | | 199 | | 185 |  |  |  |
| **D17MIT43** | | 124 | | 126/132 |  |  |  |
| **D18MIT156** | | 101 | | 108/124 |  |  |  |
| **D18Mit8** | | 75 | | 77 |  |  |  |
| **D2Mit493** | | 109 | | 127 |  |  |  |
| **D2MIT310** | | 132 | | 138/126 |  |  |  |
| **D4Mit286** | | 96 | | 76 |  |  |  |
| **D4MIT186** | | 139 | | 143/149 |  |  |  |
| **D5Mit81** | | 210 | | 194 |  |  |  |
| **D6MIT138** | | 110 | | 135 |  |  |  |
| **D6Mit59** | | 168 | | 178 |  |  |  |
| **D7Mit228** | | 148 | | 140 |  |  |  |
| **D7Mit238** | | 156 | | 132 |  |  |  |
| **D8Mit259** | | 128 | | 138 |  |  |  |
| **D8MIT271** | | 91 | | 96 |  |  |  |
| **D9Mit11** | | 74 | | 116 |  |  |  |
| **D11Mit20** | | 116 | | 150 |  |  |  |
| **D11Mit104** | | 156 | | 162 |  |  |  |
| **D14Mit121** | | 149 | | 127 |  |  |  |
| **D14MIT131** | | 107 | | 108/102 |  |  |  |
| **D17Mit202** | | 94 | | 66 |  |  |  |
| **D17MIT187** | | 242 | | 247/210 |  |  |  |
| **D19Mit46** | | 115 | | 123 |  |  |  |
| **D1Mit285** | | 129 | | 116 |  |  |  |
| **D2Mit442** | | 127 | | 105 |  |  |  |
| **D2MIT43** | | 132 | | 210/242 |  |  |  |
| **D3MIT40** | | 133 | | 103 |  |  |  |
| **D4Mit176** | | 140 | | 146 |  |  |  |
| D5Mit259 | | 140 | | 146 |  |  |  |
| **D6Mit230** | | 124 | | 138 |  |  |  |
| **D6MIT15** | | 250 | | 260/195 |  |  |  |
| **D8Mit312** | | 91 | | 83 |  |  |  |
| **D9Mit96** | | 190 | | 204 |  |  |  |
| **D9MIT184** | | 133 | | 136/130 |  |  |  |
| **D10MIT10** | | 184 | | 180/128 |  |  |  |
| **D11Mit41** | | 136 | | 166 |  |  |  |
| **D12Mit263** | | 114 | | 126 |  |  |  |
| **D13MIT213** | | 143 | | 148/160 |  |  |  |
| **D15Mit179** | | 148 | | 152 |  |  |  |
| **D15Mit70** | | 152 | | 144 |  |  |  |
| **D15MIT2** | | 85 | | 93/84 |  |  |  |
| **D16Mit58** | | 159 | | 153 |  |  |  |
| **D17Mit185** | | 190 | | 186 |  |  |  |
| **D18Mit149** | | 136 | | 116 |  |  |  |
| **D18MIT4** | | 206 | | 210/195 |  |  |  |
| **D19Mit1** | | 121 | | 142 |  |  |  |
|  | |  | |  |  |  |  |
