# Supplementary material for: A Trans-Acting Protein Effect Causes Severe Eye Malformation in the Mp Mouse
Source: PLoS Genet. 2013 Dec 12;9(12):e1003998. doi: 10.1371/journal.pgen.1003998 (PMC3861116; doi:10.1371/journal.pgen.1003998)
Supplement: Table S2 — Primary antibodies used in this study. (DOCX) [file pgen.1003998.s008.docx]

Supplemental Table S2.

| **Antigen** | **Species** | **Manufacturer/ Reference #** | **Application (Dilution)** |
| --- | --- | --- | --- |
| Fbn2 | Rabbit | L. Sakai, Portland | IHC/IF (1:300); WB (1/500) |
| Fbn1 | Rabbit | L. Sakai, Portland | IHC/IF (1:300); WB (1/500) |
| Sox2 | Rabbit | Millipore/AB5603 | IHC (1:1000) |
| Pax6 | Mouse | V. v Heyningen, Edinburgh | IHC (1:20) |
| Rhodopsin | Mouse | Abcam/ab5417 | IHC (1:2000) |
| GFAP | Mouse | Sigma/G3893 | IHC (1:2000) |
| Calbindin | Rabbit | Millipore/AB1778 | IHC (1:2000) |
| PKCα | Rabbit | Sigma/P4334 | IHC (1:2000) |
| Vsx2 | Sheep | Millipore/AB9014 | IHC (1:2000) |
| Brn3b | Goat | Santa Cruz | IHC (1:250) |
| Col6a1 | Rabbit | Tom v Agtmael, Glasgow | WB (1:100) |
| FLAG | Mouse | Sigma M2(F3165) | WB (1:500) |
| Tubulin | Mouse | Sigma B-5-1-2(T6074) | WB (1:500) |
| HSPA5(BiP) | Rabbit | CellSignal (#3177) | WB (1:1000) |
| PDI  PDI | Mouse  Rabbit | Abcam (ab2792)  CellSignal (#2446) | IF (1:250)  IHC (1:100) |
